# A Conceptual View of Cognitive Intervention in Older Adults With and Without Cognitive Decline—A Systemic Review

**DOI:** 10.3389/fragi.2022.844725

**Published:** 2022-03-24

**Authors:** Liliana Mendes, Joana Oliveira, Fernando Barbosa, Miguel Castelo-Branco

**Affiliations:** ^1^ Coimbra Institute for Clinical and Biomedical Research, Faculty of Medicine, University of Coimbra, Coimbra, Portugal; ^2^ Faculty of Psychology and Education Science, University of Porto, Porto, Portugal

**Keywords:** brain training, cognitive stimulation, cognitive training, cognitive rehabilitation, older adults

## Abstract

**Background:** Dementia is the one of the most common and prominent disease in the elderly person that results in the Cognitive interventions. In this study, we aim to conceptualize the cognitive intervention for older adults with and without cognitive dysfunction and to clarify the heterogeneity existing in this literature field by determining the main variables implicated.

**Methods:** We conducted a study analysis using previous literature highlighting the significant data reporting empirical results from cognitive intervention for healthy older adults and other seniors with different types of dementia. Each paper was reviewed in terms of compensatory cognitive training, cognitive remediation, enrichment, cognitive activation, brain training, cognitive stimulation, cognitive training, and cognitive rehabilitation. The research analysis was performed following rigorous inclusion and exclusion criteria with the purpose of collecting relevant answers to our research questions.

**Results:** We included a total of 168 studies in our review. Our findings indicated heterogeneity regarding methods, concepts, and procedures. Additionally, the values were integrated using different information existing in this field.

**Conclusion:** In conclusion, we highlighted that this is the first review that clarify the discrepancy of various existing definitions, methods, and procedures, as well as the overlapping information in the cognitive interventions.

## Introduction

According to the World Health Organization (World Health Organization, 2017b), the world’s population is rapidly aging. By 2050, it is been estimated that people over the age of 60 will account for about 22% of the world’s total population. Falls, diabetes, depression and dementia continue to be the major causes of disability in the elderly (World Health Organization, 2017). Besides musculoskeletal, sensory, immune and other disorders, cognitive functioning is also a matter of concern regarding the health of the elderly, impacting their intrinsic and functional capacity (World Health Organization, 2015b). Intrinsic capacity encompasses both physical and mental attributes people can rely on, throughout the course of their life, while functional capacity is related to having the abilities that allow them to be what they want to be (World Health Organization, 2015a). The significant increase in the average life expectancy in the last century has had consequences in this age group, namely the rapid growth of neurodegenerative dementias (Murman, 2015). Moreover, consequences have been seen at social and economic levels, particularly on both the health system and the labor market, directly affecting the elderly, who face new challenges related to cognitive deterioration (Hedden and Gabrieli, 2004).

Normal aging is a process of human development that inevitably entails biological and physiological (structural and functional) brain changes with neuropsychological and social consequences. However, the age factor may lead to different degrees of physical or mental decline (World Health Organization, 2015a). The key to healthy aging is to engage in both physically and mentally stimulating activities (Lee, 2000). Limitations in the ability to independently perform activities of daily living (ADL) are negatively associated with physical and mental well-being (Willis, 1996). In fact, the concept of ‘activities of daily living’ in the elderly is related to physical, emotional, and cognitive aspects. Cognitive aging in particular depends on intelligence, education, and sensory abilities (Drag and Bieliauskas, 2010).

The concept of healthy aging has been defined as a process of development and maintenance of functional capacity (World Health Organization, 2015) or adaptation to the physical, social, and psychological changes that allow the well-being of the elderly (Peel et al., 2004). Cognitive changes are core in this concept because of their close relationship to impairment that affects ADL and functional capacity (Yam and Marsiske, 2013). Thus, in the last decade, we have witnessed an increase in research on healthy aging and lifestyle associated with older adults’ cognitive functioning, namely, the development of neuropsychological interventions using new technologies capable of promoting the older adults’ quality of life (QoL).

There are three types of cognitive decline in the elderly: normal aging (normal cognitive decline), pre-dementia (mild cognitive deficit) (Petersen et al., 2001), and different types of dementia (severe cognitive deficit) (Alves et al., 2013). Cognitive intervention consists of various treatments based on different theoretical constructs, aimed to improve an impaired function, prevent cognitive decline, or, at least, maintain the functional level (Gates and Sachdev, 2014). Given that cognitive intervention encompasses several distinct concepts (e.g., compensatory cognitive training, cognitive remediation, cognitive training), it is important to further analyze these concepts, clarify their similarities and differences, so that these can be considered in future research. Therefore, the main aim of this study is to define the types of cognitive intervention and corresponding methodologies usually applied in older adults and clearly distinguishing the terms usually used in the literature. After reviewing the literature regarding older adults, there seem to be eight different types of cognitive interventions that are most commonly used, and main motivations are to focus on this work: Compensatory Cognitive Training, Cognitive Remediation, Enrichment, Cognitive Activation, Brain Training, Cognitive Stimulation, Cognitive Training, and Cognitive Rehabilitation.

Consequently, it is important to discuss what are the most critical differences between these distinct non-pharmacological treatments, relating to definitions, methods, and procedures.

## Studies Characteristics

**FIGURE 1:**
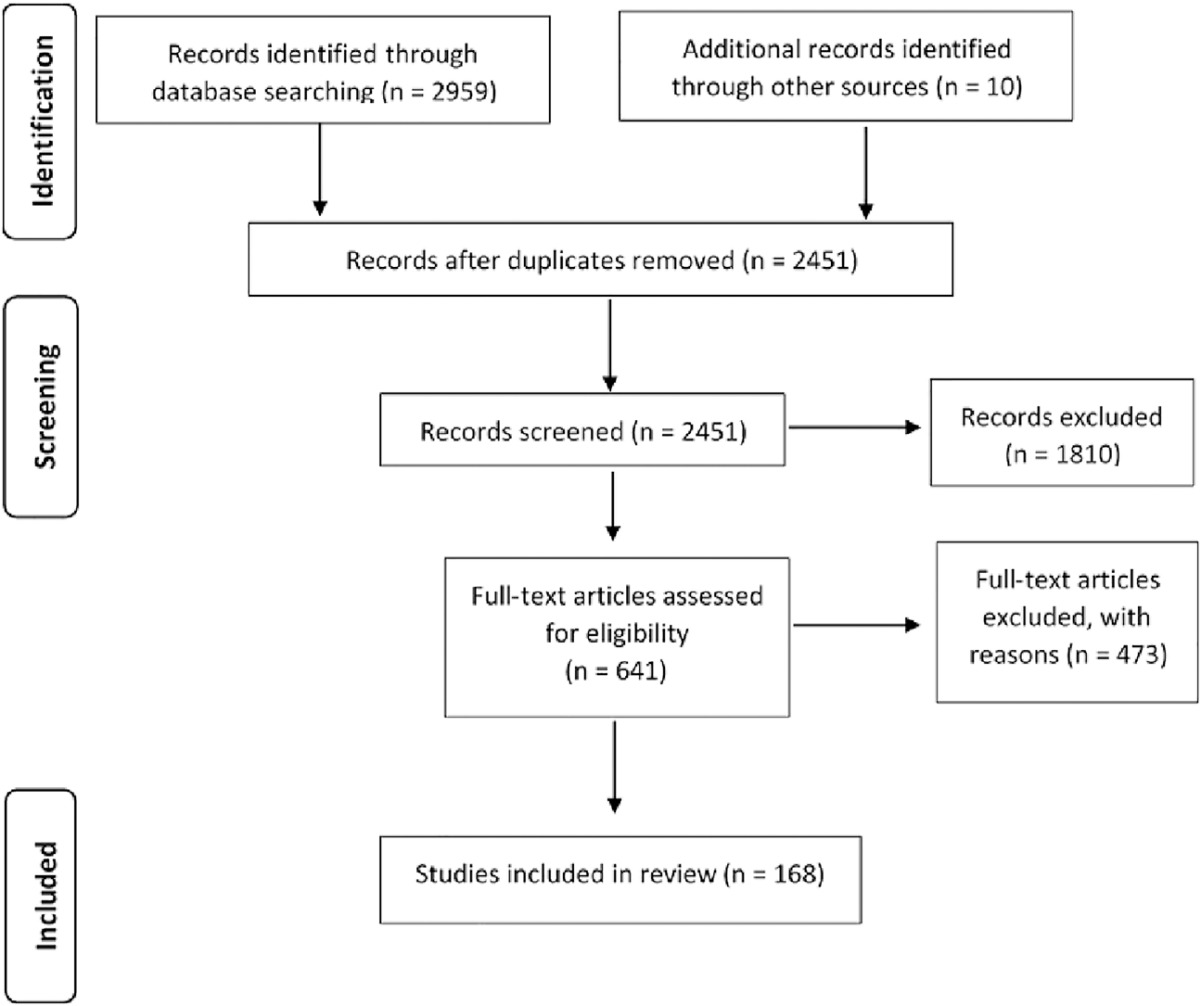
Literature search flow on cognitive interventions.

We found total 168 articles (Figure 1) through database searching (Supplementary Appendix S1) in which 59 were randomized controlled studies with post-intervention follow-up of the participants, 74 were randomized controlled trials without follow-up, 28 used a pre and post-test design with a 3–12-month post-intervention follow-up, two non-randomized pilot studies, two were descriptive and exploratory studies, two were longitudinal studies, and one was a single-blind wait list-controlled study.

Mostly in studies investigated the sample size was approximately 97 both healthy or composed healthy older adults, 26 studies were on older adults with mild cognitive impairment (MCI) or amnestic MCI (aMCI), two studies on psychotic disorders, 19 studies on older adults with dementia or mild to moderate dementia, 21 studies on Parkinson’s disease (PD), Alzheimer’s disease (AD), and older adults with cognitive impairment, and one studied stroke, HIV-associated neurocognitive disorders in older adults, and older adults with major depression.

The studies included were thoroughly reviewed in terms of the following types of cognitive intervention: Compensatory Cognitive Training, Cognitive Remediation, Enrichment, Cognitive Activation, Brain Training, Cognitive Stimulation, Cognitive Training, and Cognitive Rehabilitation (Table 1). The criteria for determining the type of cognitive intervention used in each study are summarized in Table 2. Our review includes 35 studies on Cognitive Stimulation and 11 on Cognitive Rehabilitation intervention where most of the tasks did not include computerized tasks; 102 studies on Cognitive Training intervention where most of the tasks were presented in computerized form; three studies on Enrichment, six on Cognitive Remediation and seven on Brain Training that included computer-based training or video game; and four studies on Compensatory Cognitive Training where external compensation strategies were used.

**TABLE 1:** Types of cognitive intervention defined in the literature.

Cognitive intervention	Definition	Authors
Compensatory cognitive training	Use of low-tech intervention strategies (internal, external, or environmental) to compensate for cognitive impairment, reducing its impact on activities of daily living and quality of life	Greenaway et al. (2013), Huckans et al. (2013), Kim and Kim (2014), Lenze and Bowie (2018), Twamley et al. (2012), Velligan et al. (2000)
Cognitive remediation	Neuropsychological intervention proposed by therapists for life functional and social recovery and competence development. May or not include computerized exercises to improve cognitive performance	Lenze and Bowie (2018), Medalia et al. (2002), Medalia and Richardson (2005), Medalia and Choi (2009), Mowszowski et al. (2010), Vance (2009), Vance et al. (2009)
Enrichment	All activities (activity/cognitive stimulation, social, physical, and intellectual) with a positive impact on cognitive functioning that enhance cognitive enrichment. Implementation of motor, sensory, and cognitive stimuli in the person’s environment	Hertzog et al. (2008), Steinerman (2010), Patel (2012)
Cognitive activation	Computer-based training to maintain their functioning, often with at least orientation or facilitation by people who are not therapists	Lenze and Bowie (2018)
Brain training	Is a concept mostly used by companies that commercialize to the public cognitive intervention programs. A program or activity (e.g., video games, music, computerized training, physical exercise) repeated over a period of time to improve cognitive deficits or performance in other cognitive tasks, including daily life activities (e.g., driving). brain training (Simons et al., 2016)	Heugten et al. (2016), Rabipour and Raz (2012), Simons et al. (2016)
Cognitive stimulation	Participation in activities that generally improve cognitive and social functioning. Includes multiple group activities under a social environment and multisensory stimulation	Clare and Woods (2004), Eckroth-Bucher and Siberski (2009), Gates and Sachdev (2014), Kim and Kim (2014), Kueider et al. (2014), Mowszowski et al. (2010), Steinerman (2010)
Cognitive training	Training of specific cognitive functions such as memory, executive functioning, language, and attention, involving guided practice and repetitive training	Bahar-Fuchs et al. (2013), Chaikham et al. (2016), Clare et al. (2003), Clare and Woods (2004), Eckroth-Bucher and Siberski (2009)
	Presented in paper and pencil or computerized form, with the objective of identifying, tracking, and monitoring the user	Gates et al. (2019), Kueider et al. (2014), Lenze and Bowie (2018), Mowszowski et al. (2010), Steinerman (2010)
Cognitive rehabilitation	Individualized approach, developed based on the goals of each user, i.e., in cognitive deficits, functional, and behavioral problems, and in real life. The planning and implementation of the cognitive rehabilitation plan involves the person with cognitive deficits, family members, and a set of health professionals	Bahar-Fuchs et al. (2013), Cicerone et al. (2000), Clare et al. (2003), Kueider et al. (2014), Mowszowski et al. (2010), Steinerman (2010), Wilson (2002)

**TABLE 2:** Criteria for type of cognitive intervention: compensatory cognitive training, cognitive remediation, enrichment, cognitive activation, brain training, cognitive stimulation, cognitive training, and cognitive rehabilitation.

Cognitive intervention	Characteristics	Tasks
Compensatory cognitive training	Attention, memory, executive functioning, learning of meta-cognitive strategies	External strategies such as calendar use, self-talk, note-taking, navigation devices, and a six-step problem-solving method
		Internal strategies such as mnemonic techniques, using visual imagery; using structured problem-solving, and planning methods
		Environmental strategies such as alter the workplace by removing visually distracting stimuli
Cognitive remediation	Attention, memory, processing speed, and executive function training	Computer-based cognitive training
		Cognitive-behavioral therapy and attention process training intervention; do not include computerized tasks
Enrichment	Learning, perceptual speed, memory, visuospatial skills, attention, processing speed, concentration, and executive functions	Internet-based training
		Combined physical and cognitive intervention
Brain training	Working memory, attention, memory, visuoperceptual and visuospatial skills, intelligence, language, executive functions, processing speed, and reasoning	Video games and computer-based cognitive training
Cognitive stimulation	Global cognition, fluid intelligence, executive functions, working memory, praxis, language, memory, attention, concentration, orientation, perception, processing speed, problem-solving, reasoning, and visuospatial skills	Computer-based cognitive training
		Combined physical and cognitive intervention
		Mobile and tablet-based cognitive training programs
		Cognitive stimulating activities (e.g., puzzles, quizzes, origami, autobiographical memory) that do not include computerized training
		Multiple group activities (e.g., music, poetry, museums, visual arts)
Cognitive training	Global cognition, fluid intelligence, executive functions, working memory, praxis, language, memory, attention, orientation, perception, processing speed, problem-solving, reasoning and visuospatial skills	Computer-based cognitive training
		Video games
		TV and tablet-based cognitive training programs
		Combined physical and cognitive intervention
		Combined behavioral interventions and computerized tasks
		Paper and pencil tasks
Cognitive rehabilitation	Global cognition, language, memory, executive functions, attention, concentration, problem-solving, reasoning, perception, and visuospatial abilities	Computer-based cognitive training
		Virtual environment
		Multiple group activities (e.g., self-assertiveness training, relaxation techniques, stress management, anxiety management strategies)
		Internal and external strategies such as books, diaries, post-it notes, timers, calendars that do not include computerized training
		Cognitive rehabilitation groups to train specific cognitive functions (e.g., language, memory)

## Cognitive Interventions

Cognitive Interventions (CI) is used as term to describe the variety of therapeutic approaches designed to address psychological problems at the cognitive (conscious mind) level, by the activation and analysis of thoughts, experiences, senses and memories. By using these techniques therapist helps enlist patients to develop solutions to problems going on in their mind that will be effective and permanent. Through intervention, therapist can draw the attention of the patients to the unreasonable pictures that mind has created and then begin to create tools for dealing with those pictures.

The cognitive approach to psychology assumes that your emotions, behaviors and psychology are controlled by the way you view the things that have happened around you, and cognitive interventions are launched from this point of view. In other words, if you see problems that minds create, they can be removed by changing the way of thinking and exercising your mind to work in different ways.

## Types of Cognitive Intervention

### Compensatory Cognitive Training

The literature refers to Compensatory Cognitive Training as the use of low-tech intervention strategies (internal, external, or environmental) to compensate for cognitive impairment in order to reduce its impact on ADL and QoL (Velligan et al., 2000; Twamley et al., 2012; Greenaway et al., 2013; Huckans et al., 2013; Kim and Kim, 2014; Lenze and Bowie, 2018). In general terms, it has been shown to improve memory and functionality in ADL (Greenaway et al., 2013).

This type of cognitive intervention has been implemented in various populations, including older adults, namely, individuals with aMCI, using external memory compensatory strategies (Troyer et al., 2008; Greenaway et al., 2013), and individuals with psychotic disorders (Twamley et al., 2012). Velligan et al. (2000), who tested the effect of compensatory strategies on apathy, disinhibition, and improvement of executive functions in 45 older adults with schizophrenia, indicated that patients using compensatory strategies showed greater improvement in motivation and overall functioning than those under other treatments.

However, this type of intervention’s training and applicability are controversial. It is intended for people with severe cognitive impairment and considerable functional impairment (Lenze and Bowie, 2018). Compensation focused interventions encompass cognitive interventions mainly aiming to enhance frontally mediating functions or to compensate specific cognitive functions that are adversely affected by aging. The results of a literature review by Kim and Kim (2014), which considered empirical studies with healthy, impaired, or demented older adults, indicated that Compensatory Cognitive Training is probably most effective when the intervention specifically supports frontal mediation activity and facilitates primarily compensatory adaptation in the brain according to the direction of indigenous adjustments in the aging brain.

### Cognitive Remediation

Cognitive remediation is a therapy that uses set of techniques designed to teach ‘thinking skills’ that can be thought as a form of cognitive rehabilitation. This involves training in a set of tasks that are designed to improve cognitive abilities and social functioning. The domain targeted depends upon client needs, but might include attention, working memory, planning, and executive function. However, CRT has been studied most often in schizophrenia/psychosis, but also in other conditions such as anorexia nervosa. Schizophrenia patients mainly show cognitive deficits in executive functioning, verbal fluency, and distractibility (Wykes and van der Gaag, 2001). Whereas, patients with anorexia nervosa have difficulties in set shifting tasks which are believed to correspond to cognitive inflexibility/rigid thinking that is seen in these patients group (Tchanturiaet al., 2007).

Cognitive Remediation mainly focuses on global cognitive training (e.g., theater training) or particular cognitive competencies (e.g. memory) and uses different techniques/methods of intervention, such as paper and pencil exercises, group activities, workshops, video, or computers (Vance, 2009; Vance et al., 2010). Based on a neuropsychological approach, this cognitive intervention uses a set of exercises to maintain, ameliorate, or mitigate the loss of cognition or abilities in the elderly (Vance et al., 2009). Therapists propose this type of intervention for functional and social recovery, as well as competence development (Medalia et al., 2002; Medalia and Richardson, 2005; Medalia and Choi, 2009; Vance, 2009; Vance et al., 2009; Mowszowski et al., 2010; Lenze and Bowie, 2018).

Currently, most Cognitive Remediation programs use computerized tasks to train various cognitive functions, namely, attention, memory, processing speed, and executive function (Morimoto et al., 2014). Examples are the game exercises (Sweep Seeker, Bird Safari, Target Tracker, Master Gardener, and Double decision) from the ACTIVE study (Vance et al., 2017), Mindfit (Verghese et al., 2010), and PSSCogRehab (Choi et al., 2018). Only two of the included studies followed a pre and post-test design, including cognitive-behavioral therapy and attention process training (not using computerized tasks), applied to participants with PD (Mohlman et al., 2011; Mohlman et al., 2017).

Besides the elderly, Cognitive Remediation has also been used in psychiatric populations (e.g., schizophrenia). In this case, this intervention is applied according to different theoretical perspectives, often in conjunction with psychosocial interventions or complementary to pharmacological treatment (Morimoto et al., 2012).

### Cognitive Activation

Contrary to Cognitive Remediation, the Cognitive Activation intervention is directed to healthy individuals or individuals with MCI who are autonomous and functional. Thus, therapists are not mandatory for its administration, as computer tasks are used. Moreover, there is no group intervention, and the transfer of cognitive gains to the functioning of ADL is not expected (Lenze and Bowie, 2018). Actually, the main aim of Cognitive Activation is to maintain the users’ functioning. However, for this review, we have emphasized very few results of this type of intervention on relevant literature.

### Cognitive Training

Cognitive Training involves the guided practice and repetitive training of cognitive functions using either computerized or paper and pencil methods. These cognitive intervention aims both to improve cognitive deficits (Duda and Sweet, 2020) and to identify, track, and monitor the user’s cognitive performance (Clare et al., 2003; Clare and Woods, 2004; Eckroth-Bucher and Siberski, 2009; Zelinski, 2009; Mowszowski et al., 2010; Steinerman, 2010; Bahar-Fuchs et al., 2013; Kueider et al., 2014; Chaikham et al., 2016; Lenze and Bowie, 2018; Gates et al., 2019). It has been used in persons with age-related cognitive decline (VanVleet et al., 2018), mild to moderate AD (Farina et al., 2006; Kanaan et al., 2014; Amieva et al., 2016; Giuli et al., 2016; Nousia et al., 2018; Liang et al., 2019), and loss of functionality or mild to moderate dementia, which may include structured weekly training facilitated by a therapist (De Luca et al., 2016).

Cognitive Training essentially trains specific rational functions, such as memory, executive functioning, processing speed, language and attention, through repetitive training, in older adults with or without cognitive impairment (Auffray and Juhel, 2001; Clare et al., 2003; Günther et al., 2003; Clare and Woods, 2004; Barnes et al., 2009; Eckroth-Bucher and Siberski, 2009; Irigaray et al., 2011; Zelinski et al., 2011; Cheng et al., 2012; Herrera et al., 2012; Irigaray et al., 2012; Bahar-Fuchs et al., 2013; Ball et al., 2013; Garcia-Campuzano et al., 2013; Naismith et al., 2013; Netto et al., 2013; Rebok et al., 2013; Kanaan et al., 2014; Peña et al., 2014; Rebok et al., 2014; Nouchi et al., 2016; Lee et al., 2018; Marusic et al., 2018; Smith et al., 2018; Gates et al., 2019; Matysiak et al., 2019). Previously, Kim and Kim (2014) or Thompson and Foth (2005) in most of there studies focus on memory training because problems in this cognitive domains are the most prominent concerns in the elderly.

A significant part of the studies in this review (Supplementary Appendix S1) used computerized programs to train various cognitive functions in older adults with or without cognitive alterations. Souders et al. (2017) applied the Mind Frontiers Game to 60 healthy older adults to train general reasoning, spatial reasoning, planning, processing speed, task switching and working memory. Similarly, Whitlock et al. (2012) applied the game World of Warcraft to 39 older adults to train spatial ability, executive function, and memory to detect an improvement in the intervention group’s attention. Furthermore, Basak et al. (2008) designed a video game (Rise of Nations) to train executive control functions (switching, inhibition, reasoning, working memory, short-term memory) in 40 older adults and achieved an improvement in the executive control functions. Simpson et al. (2012) also applied computerized Cognitive Training (www.mybraintrainer.com) to 34 healthy older adults, using a randomized, single-blind design with a 3-week follow-up period, and observed an improvement in reaction time, choice reaction time, and processing speed in the intervention group.

Several studies focused on the applicability of TV (Nouchi et al., 2019) and tablet-based Cognitive Training programs, either in healthy older adults (Vaportzis et al., 2017) or older adults with cognitive impairment. For example, Savulich et al. (2017) applied the Game Show to 21 older adults with MCI using a tablet and observed an improvement in episodic memory and visuospatial capacity, besides high levels of enjoyment and motivation. Chan et al. (2016) and Binder et al. (2016) also used a tablet to train cognitive functions of older adults, such as processing speed, visuospatial processing, attentional and mental control, episodic and working memory, and executive functions, and achieved an improvement in processing speed and episodic memory but no differences in mental control and visuospatial processing.

In turn, González-Palau et al. (2013) and Gonzá lez-Palau et al. (2014) used the GRADIOR software in a sample of older adults with MCI compared to healthy older adults to train perception, attention, episodic memory, and working memory. The users showed good usability and satisfaction with the intervention platform, improved verbal memory and attention, and reduced depressive symptomatology. In the same line, Fellman et al. (2020) used an internet-based platform to apply a Cognitive Training program focused on working memory in a sample of older adults with PD and verified improved working memory and reduced depression. Navarro et al. (2009) used the software How to Improve Your Mental Skills in healthy older adults to train memory and other cognitive functions and observed improved memory scores.

Other studies reinforce the use of Cognitive Training to address other cognitive domains of older adults at risk of cognitive decline. Maseda et al. (2013) developed a study on the efficacy of a Cognitive Training application (Telecognition) involving older adults with and without memory impairments. That application focused on memory, attention, language, calculation, abstract reasoning, perception, orientation and praxis. The results showed improvements in global cognitive, episodic memory, visuospatial, and verbal fluency skills on post-intervention patients without significant memory deficits. The same program was also used in older adults to address cognitive function and depressive symptomatology (Millán-Calenti et al., 2015), revealing improvement in overall cognitive functioning.

Several studies evaluated the BrainFitness program in older adults for the training of attention, working memory (Leung et al., 2015), executive function (Gooding et al., 2015) and processing speed (Smith et al., 2018; Valdés et al., 2019). Other computer-based training programs, such as CogniFit, SmartBrain, Cogmed, Lumosity, and COGPACK, have also been used to train the same cognitive functions (working memory, attention, memory and processing speed) in samples of healthy older adults (Gigler et al., 2013; Shatil, 2013) and older adults with PD (Paris et al., 2011), MCI (Belleville et al., 2006; Finn and McDonald, 2011; Vermeij et al., 2016), or subjective memory complaints (Frankenmolen et al., 2018).

Several studies applied Cognitive Training in older adults using several computerized tasks (examples are Borella et al., 2010; Wang et al., 2011; Zajac-Lamparska and Trempała, 2016; Bellander et al., 2017; Buitenweg et al., 2017; Golino et al., 2017; Grönholm-Nyman et al., 2017; Reijnders et al., 2017; Goghari and Lawlor-Savage, 2018; Withiel et al., 2019; Brum et al., 2020). Also, the results of a randomized controlled trial that included both behavioral interventions and computerized tasks—the ACTIVE Study (some examples: Ball et al., 2002; Margrett and Willis, 2006; Willis et al., 2006; Wolinsky et al., 2006; Wolinsky et al., 2009; Ball et al., 2010; Gross et al., 2011; Jones et al., 2013; Rebok et al., 2013; Sisco et al., 2013; Rebok et al., 2014; Ross et al., 2016; Ross et al., 2017; Ross et al., 2018) revealed that successful performance in daily tasks depends on executive function (Gross et al., 2011).

On the other hand, several studies applied Cognitive Training with paper and pencil or non-computerized tasks in older-adult samples with or without cognitive impairment. Some studied the Cognitive Training effects on global cognitive function in healthy older adults, which is often measured by tests that assess multiple cognitive domains (Park et al., 2009; Kwok et al., 2013; Chen et al., 2018; Rizkalla, 2018), while others focused on specific cognitive functions such as attention, working memory, language, executive functions, reasoning and visuospatial construction (Irigaray et al., 2011; Cheng et al., 2012; Irigaray et al., 2012; Jackson et al., 2012; Carretti et al., 2013; Netto et al., 2013; Feng et al., 2014; Li et al., 2016; Lopes and Argimon, 2016; Nouchi et al., 2016; Cantarella et al., 2017; Kuo et al., 2018; Rizkalla, 2018; Tagliabue et al., 2018). Regarding older adults with MCI, interventions not using computerized tasks were also focused either on global cognitive functioning (Rojas et al., 2013; Cohen-Mansfield et al., 2015; Sukontapol et al., 2018) or specific cognitive domains such as attention, memory (Cohen-Mansfield et al., 2015), language (Yan et al., 2016) and executive functions (López-Higes et al., 2018a; López-Higes et al., 2018b; López-Higes et al., 2018). In the same line, Petrelli et al. (2014) and Petrelli et al. (2015) tested the NEUROvitalis program and Peña et al. (2014) the REHACOP Cognitive Training program in older adults with PD, and both found evidence of benefits in terms of memory.

Studies on Cognitive Training have significantly increased the possibility of delaying, improving, or reversing physiological changes related to the aging process (Lustig et al., 2009; Brinke et al., 2018). However, the results of Cognitive Training are controversial, as they differ in terms of transfer effects to ADL and gains in QoL and wellbeing. Gains in the global cognitive functioning are polemic and differ with the type of population studied. However, the transfer effect seems to depend on the training time (over 30 min), its frequency (limited to one to three times a week) (Lampit et al., 2015), the age of the participants and the type of tasks (attention and working memory) (Zajac-Lamparska and Trempała, 2016). Cognitive Training may become more efficient or effective when other components satisfy compensatory function needs (Kim and Kim, 2014), as it is not always enough on its own for older adults.

### Enrichment

Some studies report the positive effect of an active lifestyle on improving overall cognitive functioning (Küster et al., 2016) in older adults without cognitive impairment or with MCI (Park and Park, 2018) and subjective memory impairments (McEwen et al., 2018). Enrichment refers to all activities (e.g., activity/cognitive, social, and physical stimulation) with a positive impact on cognitive functioning that enhance cognitive enrichment. It also comprises the implementation of motor, sensory, and cognitive stimuli in the person’s environment (Hertzog et al., 2008; Steinerman, 2010; Patel, 2012).

Different Enrichment methods are described in the literature. Linde and Alfermann (2014) developed an Enrichment intervention combining physical activity and cognitive intervention in healthy older adults. Brinke et al. (2018) applied the Fit Brains Program to older adults who received fit cognitive training, exercise and a combination of both. In the same line, Eggenberger et al. (2015) implemented a multicomponent physical exercise with simultaneous cognitive training of executive functions, episodic memory and processing speed in older adults. In turn, Best et al. (2018) combined physical, social, and cognitive enrichment using the Lumosity training program to improve cognitive function in chronic stroke. Candela et al. (2015) also applied physical activity together with the training of long-term memory and selective attention. Finally, Kalbe et al. (2018) used a specific approach involving cognitive training and physical activity with group counseling.

Enrichment can be implemented as a potential treatment for neurodegenerative diseases (Patel, 2012), after stroke (Best et al., 2018), or after traumatic brain injury (Bondi et al., 2014). Because this approach provides cognitive and physical stimulation at home, it favors an enriching environment for patients, benefiting their recovery. The older adults’ individual cognitively enriched behaviors (cognitive activity, physical exercise, among others) significantly impact their cognitive functioning improvement (Hertzog et al., 2008; Schmiedek et al., 2010).

### Cognitive Stimulation

Another technique that prevents cognitive decline is Cognitive Stimulation, which involves engaging in activities (e.g., puzzles) to improve general and social cognitive functioning (Woods et al., 2012). This approach includes multiple group activities under a social environment and multisensory stimulation (Clare and Woods, 2004; Eckroth-Bucher and Siberski, 2009; Mowszowski et al., 2010; Steinerman, 2010; Gates and Sachdev, 2014; Kim and Kim, 2014; Kueider et al., 2014).

While Cognitive Training focuses on specific cognitive domains, Cognitive Stimulation consists of engaging patients in non-specific activities to produce improvements in general mental functioning (Miranda-Castillo et al., 2012; Bahar-Fuchs et al., 2013; Herrera et al., 2017). For example, in a population with AD, the first method aims to improve, or at least maintain, cognitive and social function, while the second tries to reduce cognitive impairment (Clare and Woods, 2004; Mowszowski et al., 2010; Kueider et al., 2014). In terms of results, a meta-analysis (Liang et al., 2019) confirmed that Cognitive Training is the ideal cognitive intervention to reduce cognitive decline in people with AD, while another meta-analysis (Lin et al., 2013) concluded that Cognitive Stimulation improves the global cognitive functioning in 6–12 months in people with mild cognitive deficit or dementia.

Cognitive stimulation is applied using both computer-based and other cognitively stimulating activities (e.g., museum visits). The literature shows the usefulness of mobile applications like iBeni (Martínez-Alcalá et al., 2018), the tablet application Stim’Art (Yasini and Marchand, 2016), or video game devices such as Actively Station (Ordonez et al., 2017) and Wizard-of-Woz (Dethlefs et al., 2017) in older adults. For persons with mild to moderate cognitive impairment, the Cognitive Stimulation KODRO software can be useful to train global cognitive function, executive functions, language, working memory, processing speed, and attention (Malvy, 2016; Djabelkhir et al., 2017; Djabelkhir-Jemmi et al., 2018). Other examples using this type of intervention are the Bike Labyrinth interactive virtual bike tours, used by Karssemeijer et al. (2019) in older adults with dementia, or the Road Sign Test computer training focused on processing speed in people with MCI (Valdés et al., 2019).

In turn, most Cognitive stimulation studies that did not include computerized tasks found gains on several cognitive domains in healthy older adults (Tranter and Koutstaal, 2008; Ferná ndez-Prado et al., 2012; De Oliveira et al., 2014; Suzuki et al., 2014; Zimmermann et al., 2014; Zon et al., 2016; Grimaud et al., 2017; Herrera et al., 2017; Young, 2020) and older adults with mild to moderate dementia (Spector et al., 2003; Woods et al., 2006; Moro et al., 2012; Yamanaka et al., 2013; Moro et al., 2015; Capotosto et al., 2017; Piras et al., 2017; Stewart et al., 2017; Young et al., 2019). This intervention has also been proved to reduce cognitive decline in people with AD (Vidovich et al., 2011; Miranda-Castillo et al., 2012). Other improvements were observed in apathy and depression symptomatology (Niu et al., 2010) and conversation and communication skills (Spector et al., 2010).

Cognitive Stimulation has also been combined with physical activity. Dannhauser et al. (2014) found that this combination benefited the working memory, physical health, and fitness of older adults with MCI. Thiel et al. (2012) demonstrated that Cognitive Stimulation and physical activity might prevent age-related cognitive decline.

These positive results can be explained by the fact that Cognitive Stimulation has excellent adherence and completion rates, reasonable costs and high experiential relevance to participants, as confirmed by Alves et al. (2014) in a study involving older adults with cognitive impairment. However, Cognitive Stimulation components are controversial among authors. On one hand, Kim and Kim (2014) reported that Cognitive Stimulation includes visual, sensory, auditory, motor or social, and deep brain stimulation. On the other hand, other authors (Clare and Woods, 2004; Mowszowski et al., 2010; Steinerman, 2010; Kueider et al., 2014) describe it as involving social activities aimed to reduce cognitive decline (Newson and Kemps, 2006), improving cognitive functioning, health and well-being (Tranter and Koutstaal, 2008; Yuill and Hollis, 2011; Woods et al., 2012; Toh et al., 2016; Castel et al., 2017). Others yet report that Cognitive Stimulation can facilitate the preservation of cognitive functions, improve them with a purpose (e.g., improving attention to improve driving), or reduce the effects of aging in neurological patients (Bigand and Tillmann, 2015; Capotosto et al., 2017; Djabelkhir et al., 2017; Martínez-Alcalá et al., 2018; Rosell, 2018). This intervention also provides a non-pharmacological approach for the recovery of brain and motor functions after a disease or brain injury (Bigand and Tillmann, 2015).

### Brain Training

Brain Training is usually based on repeatedly using programs (“brain games”) focused on performance in cognitive tasks over a period of time to improve cognitive deficits. This type of intervention should include ADL such as video games, music, computerized training, or physical exercise (Rabipour and Raz, 2012; Heugten et al., 2016; Simons et al., 2016).

Some studies used Lumosity, a Brain Training platform, for the training of several cognitive domains. Ballesteros et al. (2015) and Ballesteros et al. (2017) conducted two studies focused on the effects of video games on the training of a set of cognitive functions in healthy older adults and observed improvements in the trained group in attention, working memory, processing speed, visual recognition memory, and well-being, compared to a control group. Mayas et al. (2014) also used Lumosity with older adults to train problem-solving, mental calculation, working memory, attention and confirmed the game training effects on reducing distraction and improving alertness. In turn, Nouchi et al. (2012) covered the effects of video game training with Brain Age and Tetris on executive functions and processing speed in a sample of older adults, and their results indicated transfer effects of the Brain Training game in the same cognitive domains.

Van de Ven et al. (2017) used several computerized tasks in older adults with cognitive impairment targeting executive functions, attention, reasoning, and psychomotor speed (www.braingymmer.com). Two other studies have used the Nintendo DS video games in older adults as a Brain Training method (Power et al., 2011; McDougall et al., 2012).

For some authors (McDougall and House, 2012), the older adults who benefit from Brain Training show improved perception of their cognitive functioning and QoL. However, even though there is evidence that Brain Training plays an important role in the improvement of cognitive functions, this intervention alone has no potential to achieve rehabilitation goals (namely, in functional terms) and should only be offered in combination with neuropsychological rehabilitation programs (Heugten et al., 2016).

Throughout our review, we found that the concept of Brain Training seems to be used more commercially and some researchers call it “cognitive training” (e.g., Buitenweg et al., 2017).

### Cognitive Rehabilitation

Cognitive Rehabilitation is defined as a systematic and multidisciplinary process oriented to therapeutic activities. It can follow different approaches, namely: reinforcement and reestablishment of behavior patterns; creation of new patterns of cognitive activity through the compensation of neurological deficit mechanisms, and training of compensation strategies involving the learning of cognitive skills, memory techniques, problem-solving, concentration, and critical thinking; establishment of external compensation mechanisms or environmental structuring; and adaptation and understanding of current cognitive deficits (Cicerone et al., 2000).

This intervention is an individualized approach, i.e., developed based on each user’s goals, with a comprehensive view of individual difficulties (cognitive deficits, functional and behavioral problems, and real life). The planning and implementation of Cognitive Rehabilitation involve the person with the cognitive deficit, their family members, and a team of health professionals (Cicerone et al., 2000; Wilson, 2002; Clare et al., 2003; Mowszowski et al., 2010; Steinerman, 2010; Bahar-Fuchs et al., 2013; Kueider et al., 2014).

Cognitive Rehabilitation can be focused on several cognitive domains, namely attention, concentration, memory, perception, communication, reasoning, and planning (Cicerone et al., 2000; Stuss et al., 2007). It can be computer-based or administered in a paper and pencil format (LoPresti et al., 2004; Maggio et al., 2018). Cognitive Rehabilitation has different general objectives than Cognitive Training, despite having the same approach (e.g., teaching problem-solving strategies). Specifically, Cognitive Rehabilitation uses a compensatory approach, adjusted to each person’s goals, with an intervention aiming to improve cognitive and everyday life functioning both functional and behavioral.

Cognitive Rehabilitation has been applied in a very wide range of populations, such as patients with acquired brain injury for remediation of cognitive deficits (Cicerone et al., 2000; Cicerone et al., 2005; Gordon et al., 2006; Rees et al., 2007; Rohling et al., 2009), older adults with AD (Loewenstein et al., 2004), older adults with mild cognitive deficits (Mansbach et al., 2017), and older adults with dementia (Clare et al., 2019).

Regarding the population with cognitive deficits, Vanova et al. (2018) conducted a randomized controlled trial with follow-up involving adults with MCI and mild dementia who completed a computer and internet-based program (GRADIOR and ehcoBUTLER) and observed an improvement in cognition, mood, QoL, ADL, and quality of patient-career relationship. Jelcic et al. (2014) applied the same intervention adding teleconference technology (Skype) in older adults with AD compared to a group that had face-to-face/conventional rehabilitation and a third control group that underwent face-to-face unstructured Cognitive Stimulation (e.g., practicing manual skills, reading the newspaper), and concluded that Cognitive Rehabilitation via teleconferencing improved the global cognitive functioning and language (phonemic and semantic) of people with AD. In turn, Mansbach et al. (2017) applied a computer-assisted online Cognitive Rehabilitation module (Memory Match) to 43 older adults with mild cognitive deficits and achieved an improvement in global cognition, attention, and memory in the intervention group. Another study by Kurz et al. (2009), involving older adults with MCI and AD, comprised diverse Cognitive Rehabilitation activities, namely relaxation techniques, activity planning, self-assertiveness training, stress management, external memory aids, memory training, and motor exercises.

Fasilis et al. (2018) applied a virtual environment (Main Tasks) to a group of older adults with mild dementia to train working memory, attention, problem solving, motivation, organization, impulsivity and found a relative improvement in cognitive variables. Another study by Maggio et al. (2018), with a sample of older adults with PD, used a semi-immersive therapy (virtual scenarios) system for motor and Cognitive Rehabilitation of patients with neurological diseases—BTS Nirvana—and achieved greater improvement at cognitive functioning, executive, and visuospatial abilities.

In the healthy population, Levine et al. (2007) applied a Goal Management Training program to simulate real-life tasks in healthy older adults, and the results showed an improvement in performance and self-rated executive. Craik et al. (2007) applied a program for memory training to healthy older adults that did not include computerized tasks, and there were no effects of training on working memory, primary memory, and recognition memory.

## Conclusion

To our knowledge, this is the first study to identify the various existing definitions, methods, and procedures, as well as the overlapping information regarding the cognitive intervention known including their discrepancies. This literature review aimed to discuss and characterize different types of cognitive intervention in older adults commonly reported in the relevant literature and determines what main factors may contribute to their efficacy and inefficacy. We have also seen some weaknesses in this field. Most of the studies considered (Supplementary Appendix S1) using computer-based training interventions for both healthy and neurocognitive disorders older adults, mild to moderate cognitive impairment, AD, or dementia. However, several studies did not include computerized tasks in the interventions involving healthy older adults and older adults with AD, PD, neurocognitive disorders, mild to moderate dementia, or MCI.

We also concluded that the presented studies show heterogeneity of methods regarding sample size and characteristics, outcome domains, duration and content of the intervention, number of individual treatment sessions, control condition, and main intervention. Moreover, some studies confuse certain concepts; for example, use the concept of Cognitive Stimulation or Enrichment for Cognitive Training. This inconsistency does not allow a concrete definition of the most effective intervention, its durability and the best format for older adults. Thus, it is necessary to harmonize the methodology of intervention applied to the study population.

Our conclusions should be interpreted considering some limitations. Firstly, our research was restricted to electronic databases via EBSCO (although these are the most representative and significant in the field at hand). Secondly, our attempt to contact authors and experts to access some unavailable studies was not altogether successful, but we believe that this is unlikely to have determinatively influenced our findings. Lastly, due to rigorous research with strict inclusion/exclusion criteria, we gathered a restricted pool of analyzed papers (168 studies included from 641 full-text articles assessed for eligibility). Despite these limitations, the current review allows us to find some answers to our initial research questions.

We also consider that our main purpose was achieved since a conceptualization of cognitive interventions for healthy and older adults with several types of cognitive impairments was possible. Nevertheless, a few questions remain for additional research to further enhance this non-pharmacological approach involving the elderly. In future researches, we suggest a comprehensive view to discuss the methodology of each type of intervention presented in this research and subsequently demonstrate its results and efficacy (or not) in specific older-adult populations (e.g., healthy older adults, people with different levels of cognitive decline), comparing different approaches, namely computer-based interventions and applications without computerized tasks. We also cogitate that additional studies are further needed that highlight the durability of the observed gains (or not), the transfer effects for daily functioning and well-being, the probability and risk of developing cognitive morbidity.

## References

[B1] Alves J. Alves-Costa F. Magalhães R. Gonçalves Ó. F. Sampaio A. (2014). Cognitive Stimulation for Portuguese Older Adults with Cognitive Impairment. Am. J. Alzheimers Dis. Other Demen. 29 (6), 503–512. 10.1177/1533317514522541 24526760 PMC10852816

[B2] Alves J. Magalhães R. Machado A. Gonçalves O. F. Sampaio A. Petrosyan A. (2013). Non-pharmacological Cognitive Intervention for Aging and Dementia: Current Perspectives. Wjcc 1 (8), 233–241. 10.12998/wjcc.v1.i8.233 24340275 PMC3856300

[B3] Amieva H. Robert P. H. Grandoulier A.-S. Meillon C. De Rotrou J. Andrieu S. (2016). Group and Individual Cognitive Therapies in Alzheimer's Disease: the ETNA3 Randomized Trial. Int. Psychogeriatr. 28 (5), 707–717. 10.1017/s1041610215001830 26572551

[B4] Auffray C. Juhel J. (2001). Effets généraux et différentiels d'un programme d'entraînement cognitif multimodal chez la personne âgée. psy 101 (1), 65–89. 10.3406/psy.2001.29716

[B5] Bahar-Fuchs A. Clare L. Woods B. (2013). Cognitive Training and Cognitive Rehabilitation for Mild to Moderate Alzheimer's Disease and Vascular Dementia. Cochrane Database Syst. Rev. 6, CD003260. 10.1002/14651858.CD003260.pub2 PMC7144738 23740535

[B6] Ball K. Berch D. B. Helmers K. F. Jobe J. B. Leveck M. D. Marsiske M. (2002). Effects of Cognitive Training Interventions with Older AdultsEffects of Cognitive Training Interventions with Older Adults: a Randomized Controlled Trial. JAMA 288 (18), 2271–2281. 10.1001/jama.288.18.2271 12425704 PMC2916176

[B7] Ball K. Edwards J. D. Ross L. A. McGwin Jr. G. (2010). Cognitive Training Decreases Motor Vehicle Collision Involvement of Older Drivers. J. Am. Geriatr. Soc. 58 (11), 2107–2113. 10.1111/j.1532-5415.2010.03138.x 21054291 PMC3057872

[B8] Ball K. K. Ross L. A. Roth D. L. Edwards J. D. (2013). Speed of Processing Training in the ACTIVE Study: How Much Is Needed and Who Benefits? J. Aging Health 25 (80), 65S–84S. 10.1177/0898264312470167 24385640 PMC3947605

[B9] Ballesteros S. Mayas J. Prieto A. Toril P. Pita C. Laura P. d. L. (2015). A Randomized Controlled Trial of Brain Training with Non-action Video Games in Older Adults: Results of the 3-month Follow-Up. Front. Aging Neurosci. 7, 45. 10.3389/fnagi.2015.00045 25926790 PMC4396447

[B10] Ballesteros S. Mayas J. Ruiz-Marquez E. Prieto A. Toril P. Ponce de Leon L. (2017). Effects of Video Game Training on Behavioral and Electrophysiological Measures of Attention and Memory: Protocol for a Randomized Controlled Trial. JMIR Res. Protoc. 6 (1), e8. 10.2196/resprot.6570 28119279 PMC5296621

[B11] Barnes D. E. Yaffe K. Belfor N. Jagust W. J. DeCarli C. Reed B. R. (2009). Computer-based Cognitive Training for Mild Cognitive Impairment. Alzheimer Dis. Associated Disord. 23 (3), 205–210. 10.1097/wad.0b013e31819c6137 PMC2760033 19812460

[B12] Basak C. Boot W. R. Voss M. W. Kramer A. F. (2008). Can Training in a Real-Time Strategy Video Game Attenuate Cognitive Decline in Older Adults? Psychol. Aging 23 (4), 765–777. 10.1037/a0013494 19140648 PMC4041116

[B13] Bellander M. Eschen A. Lövdén M. Martin M. Bäckman L. Brehmer Y. (2017). No Evidence for Improved Associative Memory Performance Following Process-Based Associative Memory Training in Older Adults. Front. Aging Neurosci. 8 (326), 1–11. 10.3389/fnagi.2016.00326 PMC5220050 28119597

[B14] Belleville S. Gilbert B. Fontaine F. Gagnon L. Ménard É. Gauthier S. (2006). Improvement of Episodic Memory in Persons with Mild Cognitive Impairment and Healthy Older Adults: Evidence from a Cognitive Intervention Program. Dement Geriatr. Cogn. Disord. 22, 486–499. 10.1159/000096316 17050952

[B15] Best J. R. Eng J. J. Davis J. C. Hsiung R. Hall P. A. Middleton L. E. (2018). Study Protocol for Vitality: A Proof-Of-Concept Randomised Controlled Trial of Exercise Training or Complex Mental and Social Activities to Promote Cognition in Adults with Chronic Stroke. BMJ Open 8 (3), e021490. 10.1136/bmjopen-2018-021490 PMC5875626 29550783

[B16] Bigand E. Tillmann B. (2015). “Introduction to the Neurosciences and Music V: Cognitive Stimulation and Rehabilitation,” in Annals of the New York Academy of Sciences. New York: John Wiley and Sons, 1337, vii–ix. 10.1111/nyas.12732 25773644

[B17] Binder J. C. Martin M. Zöllig J. Röcke C. Mérillat S. Eschen A. (2016). Multi-domain Training Enhances Attentional Control. Psychol. Aging 31 (4), 390–408. 10.1037/pag0000081 27294719

[B18] Bondi C. O. Klitsch K. C. Leary J. B. Kline A. E. (2014). Environmental Enrichment as a Viable Neurorehabilitation Strategy for Experimental Traumatic Brain Injury. J. Neurotrauma 31, 873–888. 10.1089/neu.2014.3328 24555571 PMC4012629

[B19] Borella E. Carretti B. Riboldi F. De Beni R. (2010). Working Memory Training in Older Adults: Evidence of Transfer and Maintenance Effects. Psychol. Aging 25 (4), 767–778. 10.1037/a0020683 20973604

[B20] Brum P. S. Borella E. Carretti B. Sanches Yassuda M. (2020). Verbal Working Memory Training in Older Adults: an Investigation of Dose Response. Aging Ment. Health 24 (1), 81–91. 10.1080/13607863.2018.1531372 30596450

[B21] Buitenweg J. Van de Ven R. Prinssen S. Murre J. Ridderinkhof K. (2017). Cognitive Flexibility Training: A Large-Scale Multimodal Adaptive Active-Control Intervention Study in Healthy Older Adults. Front. Hum. Neurosci. 11 (529), 1–14. 10.3389/fnhum.2017.00529 29209183 PMC5701641

[B22] Candela F. Zucchetti G. Magistro D. Rabaglietti E. (2015). The Effects of a Physical Activity Program and a Cognitive Training Program on the Long-Term Memory and Selective Attention of Older Adults: A Comparative Study. Activities, Adaptation & Aging 39 (1), 77–91. 10.1080/01924788.2014.977191

[B23] Cantarella A. Borella E. Carretti B. Kliegel M. de Beni R. (2017). Benefits in Tasks Related to Everyday Life Competences after a Working Memory Training in Older Adults. Int. J. Geriatr. Psychiatry 32, 86–93. 10.1002/gps.4448 26968329

[B24] Capotosto E. Belacchi C. Gardini S. Faggian S. Piras F. Mantoan V. (2017). Cognitive Stimulation Therapy in the Italian Context: its Efficacy in Cognitive and Non‐cognitive Measures in Older Adults with Dementia. Int. J. Geriatr. Psychiatry 32 (3), 331–340. 10.1002/gps.4521 27272538

[B25] Carretti B. Borella E. Zavagnin M. de Beni R. (2013). Gains in Language Comprehension Relating to Working Memory Training in Healthy Older Adults. Int. J. Geriatr. Psychiatry 28, 539–546. 10.1002/gps.3859 22821686

[B26] Castel A. Lluch C. Ribas J. Borràs L. Moltó E. (2017). Effects of a Cognitive Stimulation Program on Psychological Well-Being in a Sample of Elderly Long-Term Care Hospital Inpatients. Aging Ment. Health 21 (1), 88–94. 10.1080/13607863.2015.1099033 26496160

[B27] Chaikham A. Putthinoi S. Lersilp S. Bunpun A. Chakpitak N. (2016). Cognitive Training Program for Thai Older People with Mild Cognitive Impairment. Proced. Environ. Sci. 36, 42–45. 10.1016/j.proenv.2016.09.007

[B28] Chan M. Y. Haber S. Drew L. M. Park D. C. (2016). Training Older Adults to Use Tablet Computers: Does it Enhance Cognitive Function? Geront 56 (3), 475–484. 10.1093/geront/gnu057 PMC4873760 24928557

[B29] Chen B. Wei Y. Deng W. Sun S. (2018). The Effects of Cognitive Training on Cognitive Abilities and Everyday Function: A 10-week Randomized Controlled Trial. Int. J. Aging Hum. Dev. 86 (1), 69–81. 10.1177/0091415017697725 29212350

[B30] Cheng Y. Wu W. Feng W. Wang J. Chen Y. Shen Y. (2012). The Effects of Multi-Domain versus Single-Domain Cognitive Training in Non-demented Older People: a Randomized Controlled Trial. BMC Med. 10, 30. 10.1186/1741-7015-10-30 22453114 PMC3364144

[B31] Choi K.-H. Kang J. Kim S.-M. Lee S.-H. Park S.-C. Lee W.-H. (2018). Cognitive Remediation in Middle-Aged or Older Inpatients with Chronic Schizophrenia: A Randomized Controlled Trial in Korea. Front. Psychol. 8, 2364. 10.3389/fpsyg.2017.02364 29467684 PMC5807907

[B32] Cicerone K. D. Dahlberg C. Kalmar K. Langenbahn D. M. Malec J. F. Bergquist T. F. (2000). Evidence-based Cognitive Rehabilitation: Recommendations for Clinical Practice. Arch. Phys. Med. Rehabil. 81 (12), 1596–1615. 10.1053/apmr.2000.19240 11128897

[B33] Cicerone K. D. Dahlberg C. Malec J. F. Langenbahn D. M. Felicetti T. Kneipp S. (2005). Evidence-based Cognitive Rehabilitation: Updated Review of the Literature from 1998 through 2002. Arch. Phys. Med. Rehabil. 86 (8), 1681–1692. 10.1016/j.apmr.2005.03.024 16084827

[B34] Clare L. Woods R. T. Moniz Cook E. D. Orrell M. Spector A. (2003). Cognitive Rehabilitation and Cognitive Training for Early-Stage Alzheimer's Disease and Vascular Dementia. Cochrane Database Syst. Rev. (4), CD003260. 10.1002/14651858.CD003260 14583963

[B35] Clare L. Kudlicka A. Oyebode J. R. Jones R. W. Bayer A. Leroi I. (2019). Individual Goal‐oriented Cognitive Rehabilitation to Improve Everyday Functioning for People with Early‐stage Dementia: A Multicentre Randomised Controlled Trial (The GREAT Trial). Int. J. Geriatr. Psychiatry 34 (5), 709–721. 10.1002/gps.5076 30724405 PMC6593854

[B36] Clare L. Woods R. T. (2004). Cognitive Training and Cognitive Rehabilitation for People with Early-Stage Alzheimer's Disease: A Review. Neuropsychological Rehabil. 14 (4), 385–401. 10.1080/09602010443000074

[B37] Cohen-Mansfield J. Cohen R. Buettner L. Eyal N. Jakobovits H. Rebok G. (2015). Interventions for Older Persons Reporting Memory Difficulties: a Randomized Controlled Pilot Study. Int. J. Geriatr. Psychiatry 30 (5), 478–486. 10.1002/gps.4164 25043482

[B38] Craik F. I. Winocur G. Palmer H. Binns M. A. Edwards M. Bridges K. (2007). Cognitive Rehabilitation in the Elderly: Effects on Memory. J. Int. Neuropsychol. Soc. 13 (1), 132–142. 10.1017/S1355617707070166 17166312

[B39] Dannhauser T. M. Cleverley M. Whitfield T. J. Fletcher B. Stevens T. Walker Z. (2014). A Complex Multimodal Activity Intervention to Reduce the Risk of Dementia in Mild Cognitive Impairment-ThinkingFit: Pilot and Feasibility Study for a Randomized Controlled Trial. BMC psychiatry 14, 129. 10.1186/1471-244x-14-129 24886353 PMC4037760

[B40] De Luca R. Bramanti A. De Cola M. C. Leonardi S. Torrisi M. Aragona B. (2016). Cognitive Training for Patients with Dementia Living in a Sicilian Nursing home: a Novel Web-Based Approach. Neurol. Sci. 37 (10), 1685–1691. 10.1007/s10072-016-2659-x 27383825

[B41] De Oliveira T. C. Soares F. C. De Macedo L. D. Diniz D. L. Bento-Torres N. V. Picanço-Diniz C. W. (2014). Beneficial Effects of Multisensory and Cognitive Stimulation on Age-Related Cognitive Decline in Long-Term-Care Institutions. Clin. Interv. Aging 9, 309–320. 10.2147/CIA.S54383 24600211 PMC3933247

[B42] Dethlefs N. Milders M. Cuayáhuitl H. Al-Salkini T. Douglas L. (2017). A Natural Language-Based Presentation of Cognitive Stimulation to People with Dementia in Assistive Technology: A Pilot Study. Inform. Health Soc. Care 42 (4), 349–360. 10.1080/17538157.2016.1255627 28068146

[B43] Djabelkhir L. Wu Y. H. Vidal J. S. Cristancho-Lacroix V. Marlats F. Lenoir H. (2017). Computerized Cognitive Stimulation and Engagement Programs in Older Adults with Mild Cognitive Impairment: Comparing Feasibility, Acceptability, and Cognitive and Psychosocial Effects. Clin. Interv. Aging 12, 1967–1975. 10.2147/CIA.S145769 29200836 PMC5702161

[B44] Djabelkhir-Jemmi L. Wu Y.-H. Boubaya M. Marlats F. Lewis M. Vidal J.-S. (2018). Differential Effects of a Computerized Cognitive Stimulation Program on Older Adults with Mild Cognitive Impairment According to the Severity of white Matter Hyperintensities. Cia Vol. 13, 1543–1554. 10.2147/cia.s152225 PMC6121770 30214174

[B45] Drag L. L. Bieliauskas L. A. (2010). Contemporary Review 2009: Cognitive Aging. J. Geriatr. Psychiatry Neurol. 23 (2), 75–93. 10.1177/0891988709358590 20101069

[B46] Duda B. M. Sweet L. H. (2020). Functional Brain Changes Associated with Cognitive Training in Healthy Older Adults: A Preliminary ALE Meta-Analysis. Brain Imaging Behav. 14 (4), 1247–1262. 10.1007/s11682-019-00080-0 30900077

[B47] Eckroth-Bucher M. Siberski J. (2009). Preserving Cognition through an Integrated Cognitive Stimulation and Training Program. Am. J. Alzheimers Dis. Other Demen. 24 (3), 234–245. 10.1177/1533317509332624 19346501 PMC10845992

[B48] Eggenberger P. Schumacher V. Angst M. Theill N. de Bruin E. D. (2015). Does Multicomponent Physical Exercise with Simultaneous Cognitive Training Boost Cognitive Performance in Older Adults? A 6-month Randomized Controlled Trial with a 1-year Follow-Up. Clin. Interv. Aging 10, 1335–1349. 10.2147/CIA.S87732 26316729 PMC4544626

[B49] Farina E. Mantovani F. Fioravanti R. Pignatti R. Chiavari L. Imbornone E. (2006). Evaluating Two Group Programmes of Cognitive Training in Mild-To-Moderate AD: Is There Any Difference between a 'global' Stimulation and a 'cognitive-specific' One? Aging Ment. Health 10 (3), 211–218. 10.1080/13607860500409492 16777648

[B50] Fasilis T. Patrikelis P. Patrikelis P. Siatouni A. Alexoudi A. Veretzioti A. (2018). A Pilot Study and Brief Overview of Rehabilitation via Virtual Environment in Patients Suffering from Dementia. Psychiatriki 29 (1), 42–51. 10.22365/jpsych.2018.291.42 29754119

[B51] Fellman D. Salmi J. Ritakallio L. Ellfolk U. Rinne J. O. Laine M. (2020). Training Working Memory Updating in Parkinson's Disease: A Randomised Controlled Trial. Neuropsychological Rehabil. 30 (4), 673–708. 10.1080/09602011.2018.1489860 29968519

[B52] Feng W. Li C. Chen Y. Cheng Y. Wu W. (2014). Five-year Follow-Up Study of Multi-Domain Cognitive Training for Healthy Elderly Community Members. Shanghai Arch. Psychiatry 26 (1), 30–41. 10.3969/j.issn.1002-0829.2014.01.005 25114479 PMC4118000

[B53] Fernández-Prado S. Conlon S. Mayán-Santos J. M. Gandoy-Crego M. (2012). The Influence of a Cognitive Stimulation Program on the Quality of Life Perception Among the Elderly. Arch. Gerontol. Geriatr. 54 (1), 181–184. 10.1016/j.archger.2011.03.003 21458869

[B54] Finn M. McDonald S. (2011). Computerised Cognitive Training for Older Persons with Mild Cognitive Impairment: A Pilot Study Using a Randomised Controlled Trial Design. Brain Impairment 12 (3), 187–199. 10.1375/brim.12.3.187

[B55] Frankenmolen N. L. Overdorp E. J. Fasotti L. Claassen J. A. H. R. Kessels R. P. C. Oosterman J. M. (2018). Memory Strategy Training in Older Adults with Subjective Memory Complaints: A Randomized Controlled Trial. J. Int. Neuropsychol. Soc. 24 (10), 1110–1120. 10.1017/s1355617718000619 30168408 PMC6317111

[B56] Garcia-Campuzano M. T. Virues-Ortega J. Smith S. Moussavi Z. (2013). Effect of Cognitive Training Targeting Associative Memory in the Elderly: a Small Randomized Trial and a Longitudinal Evaluation. J. Am. Geriatr. Soc. 61 (12), 2252–2254. 10.1111/jgs.12574 24329837

[B57] Gates N. J. Rutjes A. W. Di Nisio M. Karim S. Chong L. Y. March E. (2019). Computerised Cognitive Training for Maintaining Cognitive Function in Cognitively Healthy People in Late Life. Cochrane Database Syst. Rev. 3 (3), CD012277. 10.1002/14651858.CD012277.pub2 30864187 PMC6414816

[B58] Gates N. J. Sachdev P. (2014). Is Cognitive Training an Effective Treatment for Preclinical and Early Alzheimer's Disease? J. Alzheimers Dis. 42 Suppl 4 (Suppl. 4), S551–S559. 10.3233/JAD-141302 25171716

[B59] Gigler K. L. Blomeke K. Shatil E. Weintraub S. Reber P. J. (2013). Preliminary Evidence for the Feasibility of at-home Online Cognitive Training with Older Adults. Gerontechnology 12 (1), 26–35. 10.4017/gt.2013.12.1.007.00 26778939 PMC4712702

[B60] Giuli C. Papa R. Lattanzio F. Postacchini D. (2016). The Effects of Cognitive Training for Elderly: Results from My Mind Project. Rejuvenation Res. 19 (6), 485–494. 10.1089/rej.2015.1791 26952713 PMC5178004

[B61] Goghari V. M. Lawlor-Savage L. (2018). Self-Perceived Benefits of Cognitive Training in Healthy Older Adults. Front. Aging Neurosci. 10 (112), 112–210. 10.3389/fnagi.2018.00112 29922146 PMC5996899

[B62] Golino M. T. S. Flores Mendoza C. Golino H. F. (2017). Effects of Cognitive Training on Cognitive Performance of Healthy Older Adults. Span. J. Psychol. 20, E39. 10.1017/sjp.2017.38 28929999

[B63] González-Palau F. Franco M. Bamidis P. Losada R. Parra E. Papageorgiou S. G. (2014). The Effects of a Computer-Based Cognitive and Physical Training Program in a Healthy and Mildly Cognitive Impaired Aging Sample. Aging Ment. Health 18 (7), 838–846. 10.1080/13607863.2014.899972 24697325

[B64] González-Palau F. Franco M. Toribio J. Losada R. Parra E. Bamidis P. (2013). Designing a Computer-Based Rehabilitation Solution for Older Adults: The Importance of Testing Usability. PsychNology J. 11 (2), 119–136.

[B65] Gooding A. L. Choi J. Fiszdon J. M. Wilkins K. Kirwin P. D. Van Dyck C. H. (2015). Comparing Three Methods of Computerised Cognitive Training for Older Adults with Subclinical Cognitive Decline. Neuropsychol. Rehabil. 26 (5-6), 810–821. 10.1080/09602011.2015.1118389 26674122

[B66] Gordon W. A. Cantor J. Ashman T. Brown M. (2006). Treatment of Post-TBI Executive Dysfunction. J. Head Trauma Rehabil. 21 (2), 156–167. 10.1097/00001199-200603000-00008 16569989

[B67] Greenaway M. C. Duncan N. L. Smith G. E. (2013). The Memory Support System for Mild Cognitive Impairment: Randomized Trial of a Cognitive Rehabilitation Intervention. Int. J. Geriatr. Psychiatry 28 (4), 402–409. 10.1002/gps.3838 22678947 PMC3766962

[B68] Grimaud É. Taconnat L. Clarys D. (2017). Cognitive Stimulation in Healthy Older Adults: a Cognitive Stimulation Program Using Leisure Activities Compared to a Conventional Cognitive Stimulation Program. Gériatrie Psychol. Neuropsychiatrie du Vieillissement 15 (2), 214–223. 10.1684/pnv.2017.0669 28625942

[B69] Grönholm-Nyman P. Soveri A. Rinne J. O. Ek E. Nyholm A. Stigsdotter Neely A. (2017). Limited Effects of Set Shifting Training in Healthy Older Adults. Front. Aging Neurosci. 9 (69), 69–21. 10.3389/fnagi.2017.00069 28386226 PMC5362725

[B70] Gross A. L. Rebok G. W. Unverzagt F. W. Willis S. L. Brandt J. (2011). Cognitive Predictors of Everyday Functioning in Older Adults: Results from the Active Cognitive Intervention Trial. Journals Gerontol. Ser. B: Psychol. Sci. Soc. Sci. 66B (5), 557–566. 10.1093/geronb/gbr033 PMC3155028 21558167

[B71] Günther V. Schäfer P. Holzner B. Kemmler G. (2003). Long-term Improvements in Cognitive Performance through Computer-Assisted Cognitive Training: A Pilot Study in a Residential home for Older People. Aging Ment. Health 7 (3), 200–206. 10.1080/1360786031000101175 12775401

[B72] Hedden T. Gabrieli J. D. E. (2004). Insights into the Ageing Mind: a View from Cognitive Neuroscience. Nat. Rev. Neurosci. 5 (2), 87–96. 10.1038/nrn1323 14735112

[B73] Herrera C. Chambon C. Michel B. F. Paban V. Alescio-Lautier B. (2012). Positive Effects of Computer-Based Cognitive Training in Adults with Mild Cognitive Impairment. Neuropsychologia 50, 1871–1881. 10.1016/j.neuropsychologia.2012.04.012 22525705

[B74] Hertzog C. Kramer A. F. Wilson R. S. Lindenberger U. (2008). Enrichment Effects on Adult Cognitive Development. Psychol. Sci. Public Interest 9 (1), 1–65. 10.1111/j.1539-6053.2009.01034.x 26162004

[B75] Huckans M. Hutson L. Twamley E. Jak A. Kaye J. Storzbach D. (2013). Efficacy of Cognitive Rehabilitation Therapies for Mild Cognitive Impairment (MCI) in Older Adults: Working toward a Theoretical Model and Evidence-Based Interventions. Neuropsychol. Rev. 23 (1), 63–80. 10.1007/s11065-013-9230-9 23471631 PMC3640648

[B76] Irigaray T. Filho I. Schneider R. (2012). Efeitos de um treino de atenção, memória e funções executivas na cognição de idosos saudáveis. Psicologia: Reflexão e Crítica 25 (1), 188–202. 10.1590/s0102-79722012000100023

[B77] Irigaray T. Q. Schneider R. H. Gomes I. (2011). Efeitos de um treino cognitivo na qualidade de vida e no bem-estar psicológico de idosos. Psicol. Reflex. Crit. 24 (4), 810–818. 10.1590/s0102-79722011000400022

[B78] Jackson J. J. Hill P. L. Payne B. R. Roberts B. W. Stine-Morrow E. A. L. (2012). Can an Old Dog Learn (And Want to Experience) New Tricks? Cognitive Training Increases Openness to Experience in Older Adults. Psychol. Aging 27 (2), 286–292. 10.1037/a0025918 22251379 PMC3330146

[B79] Jelcic N. Agostini M. Meneghello F. Bussè C. Parise S. Galano A. (2014). Feasibility and Efficacy of Cognitive Telerehabilitation in Early Alzheimer's Disease: a Pilot Study. Clin. Interv. Aging 9, 1605–1611. 10.2147/CIA.S68145 25284993 PMC4181448

[B80] Jones R. N. Marsiske M. Ball K. Rebok G. Willis S. L. Morris J. N. (2013). The ACTIVE Cognitive Training Interventions and Trajectories of Performance Among Older Adults. J. Aging Health 25 (0), 186S–208S. 10.1177/0898264312461938 23103453 PMC3866224

[B81] Kalbe E. Roheger M. Paluszak K. Meyer J. Becker J. Fink G. R. (2018). Effects of a Cognitive Training with and without Additional Physical Activity in Healthy Older Adults: A Follow-Up 1 Year after a Randomized Controlled Trial. Front. Aging Neurosci. 10, 407. 10.3389/fnagi.2018.00407 30618714 PMC6305338

[B82] Kanaan S. F. McDowd J. M. Colgrove Y. Burns J. M. Gajewski B. Pohl P. S. (2014). Feasibility and Efficacy of Intensive Cognitive Training in Early-Stage Alzheimer's Disease. Am. J. Alzheimers Dis. Other Demen. 29 (2), 150–158. 10.1177/1533317513506775 24667905 PMC10852592

[B83] Karssemeijer E. G. A. Aaronson J. A. Bossers W. J. R. Donders R. Olde Rikkert M. G. M. Kessels R. P. C. (2019). The Quest for Synergy between Physical Exercise and Cognitive Stimulation via Exergaming in People with Dementia: a Randomized Controlled Trial. Alz Res. Ther. 11 (1), 3. 10.1186/s13195-018-0454-z PMC6320611 30611286

[B84] Kim E. Y. Kim K. W. (2014). A Theoretical Framework for Cognitive and Non-cognitive Interventions for Older Adults: Stimulation versus Compensation. Aging Ment. Health 18 (3), 304–315. 10.1080/13607863.2013.868404 24354740

[B85] Koltai D. Welsh-Bohmer K. Schmechel D. (2001). Influence of Anosognosia on Treatment Outcome Among Dementia Patients. Neuropsychological Rehabil. 11 (3-4), 455–475. 10.1080/09602010042000097

[B86] Kueider A. Bichay K. Rebok G. (2014). “Cognitive Training for Older Adults: What Is it and Does it Work?,” in Center on Aging at American Institutes for Research. Washington: American Institutes for Research, 1–8.

[B87] Kuo C. Y. Huang Y. M. Yeh Y. Y. (2018). Let's Play Cards: Multi-Component Cognitive Training with Social Engagement Enhances Executive Control in Older Adults. Front. Psychol. 9 (2482), 2482. 10.3389/fpsyg.2018.02482 30574114 PMC6291491

[B88] Kurz A. Pohl C. Ramsenthaler M. Sorg C. (2009). Cognitive Rehabilitation in Patients with Mild Cognitive Impairment. Int. J. Geriat. Psychiatry 24, 163–168. 10.1002/gps.2086 18636436

[B89] Küster O. C. Fissler P. Laptinskaya D. Thurm F. Scharpf A. Woll A. (2016). Cognitive Change Is More Positively Associated with an Active Lifestyle Than with Training Interventions in Older Adults at Risk of Dementia: A Controlled Interventional Clinical Trial. BMC Psychiatry 16 (315), 315. 10.1186/s12888-016-1018-z 27608620 PMC5016950

[B90] Kwok T. Wong A. Chan G. Shiu Y. Y. Lam K. C. Young D. (2013). Effectiveness of Cognitive Training for Chinese Elderly in Hong Kong. Cia 8, 213–219. 10.2147/cia.s38070 PMC3578501 23440076

[B91] Lampit A. Valenzuela M. Gates N. J. (2015). Computerized Cognitive Training Is Beneficial for Older Adults. J. Am. Geriatr. Soc. 63 (12), 2610–2612. 10.1111/jgs.13825 26662712

[B92] Lee G. J. Bang H. J. Lee K. M. Kong H. H. Seo H. S. Oh M. (2018). A Comparison of the Effects between 2 Computerized Cognitive Training Programs, Bettercog and COMCOG, on Elderly Patients with MCI and Mild Dementia: A Single-Blind Randomized Controlled Study. Medicine (Baltimore) 97 (45), e13007. 10.1097/MD.0000000000013007 30407291 PMC6250524

[B93] Lee Y. (2000). The Predictive Value of Self Assessed General, Physical, and Mental Health on Functional Decline and Mortality in Older Adults. J. Epidemiol. Community Health 54 (2), 123–129. 10.1136/jech.54.2.123 10715745 PMC1731623

[B94] Lenze E. J. Bowie C. R. (2018). Cognitive Training for Older Adults: What Works? J. Am. Geriatr. Soc. 66 (4), 645–647. 10.1111/jgs.15230 29345742

[B95] Leung N. T. Y. Tam H. M. K. Chu L. W. Kwok T. C. Y. Chan F. Lam L. C. W. (2015). Neural Plastic Effects of Cognitive Training on Aging Brain. Neural Plasticity 2015, 535618. 10.1155/2015/535618 26417460 PMC4568366

[B96] Levine B. Stuss D. T. Winocur G. Binns M. A. Fahy L. Mandic M. (2007). Cognitive Rehabilitation in the Elderly: Effects on Strategic Behavior in Relation to Goal Management. J. Int. Neuropsychol. Soc. 13 (1), 143–152. 10.1017/S1355617707070178 17166313

[B97] Li B. Zhu X. Hou J. Chen T. Wang P. Li J. (2016). Combined Cognitive Training vs. Memory Strategy Training in Healthy Older Adults. Front. Psychol. 7, 834. 10.3389/fpsyg.2016.00834 27375521 PMC4896109

[B98] Liang J.-H. Li J.-Y. Jia R.-X. Wang Y.-Q. Wu R.-K. Zhang H.-B. (2019). Comparison of Cognitive Intervention Strategies for Older Adults with Mild to Moderate Alzheimer's Disease: A Bayesian Meta-Analytic Review. J. Am. Med. Directors Assoc. 20 (3), 347–355. 10.1016/j.jamda.2018.09.017 30459116

[B99] Lin J. S. O'Connor E. Rossom R. C. Perdue L. A. Eckstrom E. (2013). Screening for Cognitive Impairment in Older Adults: A Systematic Review for the U.S. Preventive Services Task Force. Ann. Intern. Med. 159 (9), 601–612. 10.7326/0003-4819-159-9-201311050-00730 24145578

[B100] Linde K. Alfermann D. (2014). Single versus Combined Cognitive and Physical Activity Effects on Fluid Cognitive Abilities of Healthy Older Adults: a 4-month Randomized Controlled Trial with Follow-Up. J. Aging Phys. activity 22 (3), 302–313. 10.1123/japa.2012-0149 23881448

[B101] Loewenstein D. A. Acevedo A. Czaja S. J. Duara R. (2004). Cognitive Rehabilitation of Mildly Impaired Alzheimer Disease Patients on Cholinesterase Inhibitors. Am. J. Geriatr. Psychiatry 12 (4), 395–402. 10.1097/00019442-200407000-00007 15249277

[B102] Lopes R. M. F. Argimon I. I. d. L. (2016). El entrenamiento cognitivo en los ancianos y efectos en las funciones ejecutivas. Act.Colom.Psicol. 19 (2), 159–176. 10.14718/acp.2016.19.2.8

[B103] López-Higes R. Martín-Aragoneses M. Rubio-Valdehita S. Delgado-Losada M. Montejo P. Montenegro M. (2018a). Efficacy of Cognitive Training in Older Adults with and without Subjective Cognitive Decline Is Associated with Inhibition Efficiency and Working Memory Span, Not with Cognitive reserve. Front. Aging Neurosci. 10 (23). 10.3389/fnagi.2018.00023 PMC5801297 29456502

[B104] López-Higes R. Prados J. Rubio-Valdehita S. Rodríguez-Rojo I. Frutos-Lucas J. Montenegro M. (2018b). Factors Explaining Language Performance after Training in Elders with and without Subjective Cognitive Decline. Front. Aging Neurosci. 10 (264). 10.3389/fnagi.2018.00264 PMC6129583 30233353

[B105] LoPresti E. F. Mihailidis A. Kirsch N. (2004). Assistive Technology for Cognitive Rehabilitation: State of the Art. Neuropsychological Rehabil. 14 (1/2), 5–39. 10.1080/09602010343000101

[B106] Lustig C. Shah P. Seidler R. Reuter-Lorenz P. A. (2009). Aging, Training, and the Brain: A Review and Future Directions. Neuropsychol. Rev. 19 (4), 504–522. 10.1007/s11065-009-9119-9 19876740 PMC3005345

[B107] Maggio M. G. De Cola M. C. Latella D. Maresca G. Finocchiaro C. La Rosa G. (2018). What about the Role of Virtual Reality in Parkinson Disease's Cognitive Rehabilitation? Preliminary Findings from a Randomized Clinical Trial. J. Geriatr. Psychiatry Neurol. 31 (6), 312–318. 10.1177/0891988718807973 30360679

[B108] Malvy L. (2016). Bénéfice de l'utilisation d'un programme de stimulation cognitive sur tablettes tactiles chez des personnes âgées dépendantes et institutionnalisées : programme KODRO. NPG Neurologie - Psychiatrie - Gériatrie 16, 344–352. 10.1016/j.npg.2016.02.006

[B109] Mansbach W. E. Mace R. A. Clark K. M. (2017). The Efficacy of a Computer-Assisted Cognitive Rehabilitation Program for Patients with Mild Cognitive Deficits: A Pilot Study. Exp. Aging Res. 43 (1), 94–104. 10.1080/0361073x.2017.1258256 28067610

[B110] Margrett J. A. Willis S. L. (2006). In-home Cognitive Training with Older Married Couples: Individual versus Collaborative Learning. Aging Neuropsychol. CognitionSection B, Aging Neuropsychol. Cogn. 13 (2), 173–195. 10.1080/138255890969285 PMC2856450 16807197

[B111] Martínez-Alcalá C. Rosales-Lagarde A. Hernández-Alonso E. Melchor-Agustin R. Rodriguez-Torres E. Itzá-Ortiz B. (2018). A mobile App (iBeni) with a Neuropsychological Basis for Cognitive Stimulation for Elderly Adults: Pilot and Validation Study. JMIR Res. Protoc. 7 (8), e172. 10.2196/resprot.9603 30131319 PMC6123536

[B112] Marusic U. Giordani B. Moffat S. D. Petrič M. Dolenc P. Pišot R. (2018). Computerized Cognitive Training during Physical Inactivity Improves Executive Functioning in Older Adults. Aging Neuropsychol. Cogn. 25 (1), 49–69. 10.1080/13825585.2016.1263724 27937138

[B113] Maseda A. Millán-Calenti J. C. Lorenzo-López L. Núñez-Naveira L. (2013). Efficacy of a Computerized Cognitive Training Application for Older Adults with and without Memory Impairments. Aging Clin. Exp. Res. 25 (4), 411–419. 10.1007/s40520-013-0070-5 23780693

[B114] Matysiak O. Kroemeke A. Brzezicka A. (2019). Working Memory Capacity as a Predictor of Cognitive Training Efficacy in the Elderly Population. Front. Aging Neurosci. 11 (126), 126. 10.3389/fnagi.2019.00126 31214015 PMC6554703

[B115] Mayas J. Parmentier F. B. R. Andrés P. Ballesteros S. (2014). Plasticity of Attentional Functions in Older Adults after Non-action Video Game Training: a Randomized Controlled Trial. PLoS One 9 (3), e92269. 10.1371/journal.pone.0092269 24647551 PMC3960226

[B116] McDougall S. House B. (2012). Brain Training in Older Adults: Evidence of Transfer to Memory Span Performance and Pseudo-matthew Effects. Neuropsychol. Dev. Cogn. B Aging Neuropsychol. Cogn. 19 (1-2), 195–221. 10.1080/13825585.2011.640656 22248429

[B117] McEwen S. C. Siddarth P. Abedelsater B. Kim Y. Mui W. Wu P. (2018). Simultaneous Aerobic Exercise and Memory Training Program in Older Adults with Subjective Memory Impairments. Jad 62 (2), 795–806. 10.3233/jad-170846 29480182 PMC5870016

[B118] Medalia A. Revheim N. Casey M. (2002). Remediation of Problem-Solving Skills in Schizophrenia: Evidence of a Persistent Effect. Schizophr Res. 57 (2-3), 165–171. 10.1016/s0920-9964(01)00293-6 12223247

[B119] Medalia A. Choi J. (2009). Cognitive Remediation in Schizophrenia. Neuropsychol. Rev. 19 (3), 353–364. 10.1007/s11065-009-9097-y 19444614

[B120] Medalia A. Richardson R. (2005). What Predicts a Good Response to Cognitive Remediation Interventions? Schizophrenia Bull. 31 (4), 942–953. 10.1093/schbul/sbi045 16120830

[B121] Melguizo Herrera E. Bertel De La Hoz A. Paternina Osorio D. Felfle Fuentes Y. Porto Osorio L. (2017). Cognitive Stimulation of Elderly Residents in Social Protection Centers in Cartagena, 2014. Revista Colombiana de Psiquiatría 46 (4), 229–236. 10.1016/j.rcp.2016.09.008 29122230

[B122] Millán-Calenti J. C. Lorenzo T. Núñez-Naveira L. Buján A. Rodríguez-Villamil J. L. Maseda A. (2015). Efficacy of a Computerized Cognitive Training Application on Cognition and Depressive Symptomatology in a Group of Healthy Older Adults: A Randomized Controlled Trial. Arch. Gerontol. Geriatr. 61 (3), 337–343. 26321734 10.1016/j.archger.2015.08.015

[B123] Miranda-Castillo C. Tapia F. Herrera A. Ghigliotto F. Guerra L. (2012). Implementación de un programa de estimulación cognitiva en personas con demencia tipo Alzheimer: Un estudio piloto en chilenos de la tercera edad. Universitas Psychologica 12 (2), 445–455.

[B124] Mohlman J. Chazin D. Georgescu B. (2011). Feasibility and Acceptance of a Nonpharmacological Cognitive Remediation Intervention for Patients with Parkinson Disease. J. Geriatr. Psychiatry Neurol. 24 (2), 91–97. 10.1177/0891988711402350 21546649

[B125] Mohlman J. DeVito A. Lauderdale S. Dobkin R. (2017). Initial Outcomes of a Combined Cognitive-Behavioral Therapy and Attention Process Training Intervention for Older Adults with Parkinson's Disease. Pract. Innov. 2 (4), 234–242. 10.1037/pri0000056

[B126] Morimoto S. S. Wexler B. E. Alexopoulos G. S. (2012). Neuroplasticity-based Computerized Cognitive Remediation for Geriatric Depression. Int. J. Geriatr. Psychiatry 27 (12), 1239–1247. 10.1002/gps.3776 22451346 PMC3387346

[B127] Morimoto S. S. Wexler B. E. Liu J. Hu W. Seirup J. Alexopoulos G. S. (2014). Neuroplasticity-based Computerized Cognitive Remediation for Treatment-Resistant Geriatric Depression. Nat. Commun. 5, 4579. 10.1038/ncomms5579 25093396 PMC4139707

[B128] Moro V. Condoleo M. T. Sala F. Pernigo S. Moretto G. Gambina G. (2012). Cognitive Stimulation in A-MCI. Am. J. Alzheimers Dis. Other Demen. 27 (2), 121–130. 10.1177/1533317512441386 22495340 PMC10697381

[B129] Moro V. Condoleo M. T. Valbusa V. Broggio E. Moretto G. Gambina G. (2015). Cognitive Stimulation of Executive Functions in Mild Cognitive Impairment. Am. J. Alzheimers Dis. Other Demen. 30 (2), 153–164. 10.1177/1533317514539542 24963080 PMC10852843

[B130] Mowszowski L. Batchelor J. Naismith S. L. (2010). Early Intervention for Cognitive Decline: Can Cognitive Training Be Used as a Selective Prevention Technique? Int. Psychogeriatr. 22 (4), 537–548. 10.1017/s1041610209991748 20170585

[B131] Murman D. L. (2015). The Impact of Age on Cognition. Semin. Hear. 36 (3), 111–121. 10.1055/s-0035-1555115 27516712 PMC4906299

[B132] Naismith S. L. Mowszowski L. Diamond K. Lewis S. J. G. (2013). Improving Memory in Parkinson's Disease: A Healthy Brain Ageing Cognitive Training Program. Mov Disord. 28 (8), 1097–1103. 10.1002/mds.25457 23630134

[B133] Navarro J. Menacho I. Alcalde C. Marchena E. Ruiz G. Aguilar M. (2009). Cognitive Changes Among Institutionalized Elderly People. Uedg 35 (6), 523–540. 10.1080/03601270802608568

[B134] Netto T. M. Greca D. V. Zimmermann N. Oliveira C. R. d. Teixeira-Leite H. M. Fonseca R. P. (2013). Efeito de um programa de treinamento da memória de trabalho em adultos idosos. Psicol. Reflex. Crit. 26 (1), 122–135. 10.1590/s0102-79722013000100014

[B135] Newson R. S. Kemps E. B. (2006). The Influence of Physical and Cognitive Activities on Simple and Complex Cognitive Tasks in Older Adults. Exp. Aging Res. 32 (3), 341–362. 10.1080/03610730600699134 16754471

[B136] Niu Y.-X. Tan J.-P. Guan J.-Q. Zhang Z.-Q. Wang L.-N. (2010). Cognitive Stimulation Therapy in the Treatment of Neuropsychiatric Symptoms in Alzheimer's Disease: a Randomized Controlled Trial. Clin. Rehabil. 24 (12), 1102–1111. 10.1177/0269215510376004 20713437

[B137] Nouchi R. Taki Y. Takeuchi H. Hashizume H. Akitsuki Y. Shigemune Y. (2012). Brain Training Game Improves Executive Functions and Processing Speed in the Elderly: A Randomized Controlled Trial. PLoS ONE 7 (1), e29676. 10.1371/journal.pone.0029676 22253758 PMC3256163

[B138] Nouchi R. Taki Y. Takeuchi H. Nozawa T. Sekiguchi A. Kawashima R. (2016). Reading Aloud and Solving Simple Arithmetic Calculation Intervention (Learning Therapy) Improves Inhibition, Verbal Episodic Memory, Focus Attention and Processing Speed in Healthy Elderly People: Evidence from a Randomized Controlled Trial. Front. Hum. Neurosci. 10 (217), 1–14. 10.3389/fnhum.2016.00217 27242481 PMC4868921

[B139] Nouchi R. Kobayashi A. Nouchi H. Kawashima R. (2019). Newly Developed Tv-Based Cognitive Training Games Improve Car Driving Skills, Cognitive Functions, and Mood in Healthy Older Adults: Evidence from a Randomized Controlled Trial. Front. Aging Neurosci. 11 (99), 1–15. 10.3389/fnagi.2019.00099 31133842 PMC6513888

[B140] Nousia A. Siokas V. Aretouli E. Messinis L. Aloizou A.-M. Martzoukou M. (2018). Beneficial Effect of Multidomain Cognitive Training on the Neuropsychological Performance of Patients with Early-Stage Alzheimer's Disease. Neural Plasticity 2018 (2845176), 1–9. 10.1155/2018/2845176 PMC6079404 30123243

[B141] Ordonez T. N. Borges F. Kanashiro C. S. Santos C. C. d. N. Hora S. S. Lima-Silva T. B. (2017). Actively Station: Effects on Global Cognition of Mature Adults and Healthy Elderly Program Using Eletronic Games. Dement. Neuropsychol. 11 (2), 186–197. 10.1590/1980-57642016dn11-020011 29213510 PMC5710687

[B142] París A. P. Saleta H. G. de la Cruz Crespo Maraver M. Silvestre E. Freixa M. G. Torrellas C. P. (2011). Blind Randomized Controlled Study of the Efficacy of Cognitive Training in Parkinson's Disease. Mov. Disord. 26 (7), 1251–1258. 10.1002/mds.23688 21442659

[B143] Park J.-H. Park J.-H. (2018). Does Cognition-specific Computer Training Have Better Clinical Outcomes Than Non-specific Computer Training? A Single-Blind, Randomized Controlled Trial. Clin. Rehabil. 32 (2), 213–222. 10.1177/0269215517719951 28726492

[B144] Park M. H. Kwon D. Y. Seo W. K. Lim K. S. Song M. S. (2009). The Effects of Cognitive Training on Community-Dwelling Elderly Koreans. J. Psychiatr. Ment. Health Nurs. 16 (10), 904–909. 10.1111/j.1365-2850.2009.01467.x 19930364

[B145] Patel T. R. (2012). Environmental Enrichment: Aging and Memory. Yale J. Biol. Med. 85 (4), 491–500. 23239950 PMC3516891

[B146] Peel N. Bartlett H. McClure R. (2004). Healthy Ageing: How Is it Defined and Measured? Australas. J. Ageing 23 (3), 115–119. 10.1111/j.1741-6612.2004.00035.x

[B147] Peña J. Ibarretxe-Bilbao N. García-Gorostiaga I. Gomez-Beldarrain M. A. Díez-Cirarda M. Ojeda N. (2014). Improving Functional Disability and Cognition in Parkinson Disease: Randomized Controlled Trial. Neurology 83 (23), 2167–2174. 10.1212/WNL.0000000000001043 25361785 PMC4276404

[B148] Petersen R. C. Doody R. Kurz A. Mohs R. C. Morris J. C. Rabins P. V. (2001). Current Concepts in Mild Cognitive Impairment. Arch. Neurol. 58 (12), 1985–1992. 10.1001/archneur.58.12.1985 11735772

[B149] Petersen R. C. (2004). Mild Cognitive Impairment as a Diagnostic Entity. J. Intern. Med. 256 (3), 183–194. 10.1111/j.1365-2796.2004.01388.x 15324362

[B150] Petrelli A. Kaesberg S. Barbe M. T. Timmermann L. Fink G. R. Kessler J. (2014). Effects of Cognitive Training in Parkinson's Disease: a Randomized Controlled Trial. Parkinsonism Relat. Disord. 20 (11), 1196–1202. 10.1016/j.parkreldis.2014.08.023 25242806

[B151] Petrelli A. Kaesberg S. Barbe M. T. Timmermann L. Rosen J. B. Fink G. R. (2015). Cognitive Training in Parkinson's Disease Reduces Cognitive Decline in the Long Term. Eur. J. Neurol. 22 (4), 640–647. 10.1111/ene.12621 25534579

[B152] Piras F. Carbone E. Faggian S. Salvalaio E. Gardini S. Borella E. (2017). Efficacy of Cognitive Stimulation Therapy for Older Adults with Vascular Dementia. Dement. Neuropsychol. 11 (4), 434–441. 10.1590/1980-57642016dn11-040014 29354225 PMC5770003

[B153] Power K. Kirwan G. Palmer M. (2011). A Comparison of Text and Technology Based Training Tools to Improve Cognitive Skills in Older Adults. Stud. Health Technol. Inform. 167, 98–102. 21685649

[B154] Rabipour S. Raz A. (2012). Training the Brain: Fact and Fad in Cognitive and Behavioral Remediation. Brain Cogn. 79, 159–179. 10.1016/j.bandc.2012.02.006 22463872

[B155] Rebok G. W. Langbaum J. B. Jones R. N. Gross A. L. Parisi J. M. Spira A. P. (2013). Memory Training in the ACTIVE Study: How Much Is Needed and Who Benefits? J. Aging Health 25 (0), 21S–42S. 10.1177/0898264312461937 23103452 PMC3825774

[B156] Rebok G. W. Ball K. Guey L. T. Jones R. N. Kim H.-Y. King J. W. (2014). Ten-Year Effects of the Advanced Cognitive Training for Independent and Vital Elderly Cognitive Training Trial on Cognition and Everyday Functioning in Older Adults. J. Am. Geriatr. Soc. 62 (1), 16–24. 10.1111/jgs.12607 24417410 PMC4055506

[B157] Rees L. Marshall S. Hartridge C. Mackie D. Weiser M. (2007). Cognitive Interventions post Acquired Brain Injury. Brain Inj. 21 (2), 161–200. 10.1080/02699050701201813 17364530

[B158] Reijnders J. S. A. M. Geusgens C. A. V. Ponds R. W. H. M. Van Boxtel M. P. J. (2017). "Keep Your Brain Fit!" Effectiveness of a Psychoeducational Intervention on Cognitive Functioning in Healthy Adults: A Randomised Controlled Trial. Neuropsychological Rehabil. 27 (4), 455–471. 10.1080/09602011.2015.1090458 26414279

[B159] Rizkalla M. N. (2018). Cognitive Training in the Elderly: A Randomized Trial to Evaluate the Efficacy of a Self-Administered Cognitive Training Program. Aging Ment. Health 22 (10), 1384–1394. 10.1080/13607863.2015.1118679 26644269

[B160] Rohling M. L. Faust M. E. Beverly B. Demakis G. (2009). Effectiveness of cognitive rehabilitation following acquired brain injury: a meta-analytic re-examination of Cicerone et al.'s (2000, 2005) systematic reviews. Neuropsychology 23 (1), 20–39. 10.1037/a0013659 19210030

[B161] Rojas G. J. Villar V. Iturry M. Harris P. Serrano C. M. Herrera J. A. (2013). Efficacy of a Cognitive Intervention Program in Patients with Mild Cognitive Impairment. Int. Psychogeriatr. 25 (5), 825–831. 10.1017/s1041610213000045 23414646

[B162] Rosell J. (2018). Cognitive stimulation for healthy older adults through computer-based programs: A review of the literature/Estimulación cognitiva para personas mayores sanas mediante programas computarizados: Una revisión de la literatura. Estudios de Psicología 39 (2-3), 407–436. 10.1080/02109395.2018.1494678

[B163] Ross L. A. Freed S. A. Edwards J. D. Phillips C. B. Ball K. (2017). The Impact of Three Cognitive Training Programs on Driving Cessation across 10 years: A Randomized Controlled Trial. Gerontologist 57 (5), 838–846. 10.1093/geront/gnw143 28329859 PMC5881723

[B164] Ross L. A. Edwards J. D. O’Connor M. L. Ball K. K. Wadley V. G. Vance D. E. (2016). The Transfer of Cognitive Speed of Processing Training to Older Adults' Driving Mobility across 5 Years. Geronb 71 (1), 87–97. 10.1093/geronb/gbv022 PMC4701127 25878053

[B165] Ross L. A. Sprague B. N. Phillips C. B. O’Connor M. L. Dodson J. E. (2018). The Impact of Three Cognitive Training Interventions on Older Adults' Physical Functioning across 5 Years. J. Aging Health 30 (3), 475–498. 10.1177/0898264316682916 28553791 PMC5453841

[B166] Savulich G. Piercy T. Fox C. Suckling J. Rowe J. B. O’Brien J. T. (2017). Cognitive Training Using a Novel Memory Game on an Ipad in Patients with Amnestic Mild Cognitive Impairment (aMCI). Int. J. Neuropsychopharmacol. 20 (8), 624–633. 10.1093/ijnp/pyx040 28898959 PMC5569993

[B167] Schmiedek F. Bauer C. Lövdén M. Brose A. Lindenberger U. (2010). Cognitive Enrichment in Old Age. GeroPsych 23 (2), 59–67. 10.1024/1662-9647/a000013

[B168] Shatil E. (2013). Does Combined Cognitive Training and Physical Activity Training Enhance Cognitive Abilities More Than Either Alone? A Four-Condition Randomized Controlled Trial Among Healthy Older Adults. Front. Aging Neurosci. 5 (8), 8–12. 10.3389/fnagi.2013.00008 23531885 PMC3607803

[B169] Simons D. J. Boot W. R. Charness N. Gathercole S. E. Chabris C. F. Hambrick D. Z. (2016). Do "brain-training" Programs Work? Psychol. Sci. Public Interest 17 (3), 103–186. 10.1177/1529100616661983 27697851

[B170] Simpson T. Camfield D. Pipingas A. Macpherson H. Stough C. (2012). Improved Processing Speed: Online Computer-Based Cognitive Training in Older Adults. Educ. Gerontol. 38 (7), 445–458. 10.1080/03601277.2011.559858

[B171] Sisco S. M. Marsiske M. Gross A. L. Rebok G. W. (2013). The Influence of Cognitive Training on Older Adults' Recall for Short Stories. J. Aging Health 25 (80), 230S–48S. 10.1177/0898264313501386 24385636 PMC3882333

[B172] Smith M. Jones M. P. Dotson M. M. Wolinsky F. D. (2018). Speed-of-Processing Training in Assisted and Independent Living: A Randomized Controlled Trial. J. Am. Geriatr. Soc. 66 (8), 1538–1545. 10.1111/jgs.15423 29972593 PMC6133746

[B173] Souders D. J. Boot W. R. Blocker K. Vitale T. Roque N. A. Charness N. (2017). Evidence for Narrow Transfer after Short-Term Cognitive Training in Older Adults. Front. Aging Neurosci. 9, 41. 10.3389/fnagi.2017.00041 28293188 PMC5328998

[B174] Spector A. Orrell M. Woods B. (2010). Cognitive Stimulation Therapy (CST): Effects on Different Areas of Cognitive Function for People with Dementia. Int. J. Geriat. Psychiatry 25 (12), 1253–1258. 10.1002/gps.2464 20069533

[B175] Spector A. Thorgrimsen L. Woods B. Royan L. Davies S. Butterworth M. (2003). Efficacy of an Evidence-Based Cognitive Stimulation Therapy Programme for People with Dementia. Br. J. Psychiatry 183, 248–254. 10.1192/bjp.183.3.248 12948999

[B176] Steinerman J. R. (2010). Minding the Aging Brain: Technology-Enabled Cognitive Training for Healthy Elders. Curr. Neurol. Neurosci. Rep. 10 (5), 374–380. 10.1007/s11910-010-0124-4 20544402

[B177] Stewart D. B. Berg-Weger M. Tebb S. Sakamoto M. Roselle K. Downing L. (2017). Making a Difference: A Study of Cognitive Stimulation Therapy for Persons with Dementia. J. Gerontological Soc. Work 60 (4), 300–312. 10.1080/01634372.2017.1318196 28409672

[B178] Stuss D. T. Robertson I. H. Craik F. I. Levine B. Alexander M. P. Black S. (2007). Cognitive Rehabilitation in the Elderly: a Randomized Trial to Evaluate a New Protocol. J. Int. Neuropsychol. Soc. 13 (1), 120–131. 10.1017/S1355617707070154 17166311

[B179] Sukontapol C. Kemsen S. Chansirikarn S. Nakawiro D. Kuha O. Taemeeyapradit U. (2018). The Effectiveness of a Cognitive Training Program in People with Mild Cognitive Impairment: A Study in Urban Community. Asian J. Psychiatry 35, 18–23. 10.1016/j.ajp.2018.04.028 29723721

[B180] Suzuki H. Kuraoka M. Yasunaga M. Nonaka K. Sakurai R. Takeuchi R. (2014). Cognitive Intervention through a Training Program for Picture Book reading in Community-Dwelling Older Adults: a Randomized Controlled Trial. BMC Geriatr. 14, 122. 10.1186/1471-2318-14-122 25416537 PMC4247720

[B181] Tagliabue C. F. Guzzetti S. Gualco G. Boccolieri G. Boccolieri A. Smith S. (2018). A Group Study on the Effects of a Short Multi-Domain Cognitive Training in Healthy Elderly Italian People. BMC Geriatr. 18 (321), 1–11. 10.1186/s12877-018-1014-x 30587151 PMC6307149

[B230] Tchanturia K. Davies H. Campbell I. C. (2007). Cognitive Remediation Therapy for Patients with Anorexia Nervosa: Preliminary Findings. Annals of General Psychiatry 6, 14. 10.1186/1744-859X-6-14 17550611 PMC1892017

[B182] Ten Brinke L. F. Best J. R. Crockett R. A. Liu-Ambrose T. (2018). The Effects of an 8-week Computerized Cognitive Training Program in Older Adults: A Study Protocol for a Randomized Controlled Trial. BMC Geriatr. 18 (31), 31–11. 10.1186/s12877-018-0730-6 29378515 PMC5789628

[B183] Thiel C. Vogt L. Tesky V. A. Meroth L. Jakob M. Sahlender S. (2012). Cognitive Intervention Response Is Related to Habitual Physical Activity in Older Adults. Aging Clin. Exp. Res. 24 (1), 47–55. 10.3275/7569 21406956

[B184] Thompson G. Foth D. (2005). Cognitive-training Programs for Older Adults: What Are They and Can They Enhance Mental Fitness? Educ. Gerontol. 31 (8), 603–626. 10.1080/03601270591003364

[B185] Toh H. M. Ghazali S. E. Subramaniam P. (2016). The Acceptability and Usefulness of Cognitive Stimulation Therapy for Older Adults with Dementia: A Narrative Review. Int. J. Alzheimer's Dis. 2016 (2), 1–11. 10.1155/2016/5131570 PMC4958464 27478677

[B186] Tranter L. J. Koutstaal W. (2008). Age and Flexible Thinking: An Experimental Demonstration of the Beneficial Effects of Increased Cognitively Stimulating Activity on Fluid Intelligence in Healthy Older Adults. Aging Neuropsychol. Cogn. 15 (2), 184–207. 10.1080/13825580701322163 17851980

[B187] Troyer A. K. Murphy K. J. Anderson N. D. Moscovitch M. Craik F. I. M. (2008). Changing Everyday Memory Behaviour in Amnestic Mild Cognitive Impairment: a Randomised Controlled Trial. Neuropsychological Rehabil. 18 (1), 65–88. 10.1080/09602010701409684 17943615

[B188] Twamley E. W. Vella L. Burton C. Z. Heaton R. K. Jeste D. V. (2012). Compensatory Cognitive Training for Psychosis: Effects in a Randomized Controlled Trial. J. Clin. Psychiatry 73 (9), 1212–1219. 10.4088/JCP.12m07686 22939029 PMC3593661

[B189] Valdés E. G. Andel R. Lister J. J. Gamaldo A. Edwards J. D. (2019). Can Cognitive Speed of Processing Training Improve Everyday Functioning Among Older Adults with Psychometrically Defined Mild Cognitive Impairment? J. Aging Health 31 (4), 595–610. 10.1177/0898264317738828 29254421 PMC11034754

[B190] Van de Ven R. M. Buitenweg J. I. Schmand B. Veltman D. J. Aaronson J. A. Nijboer T. C. (2017). Brain Training Improves Recovery after Stroke but Waiting List Improves Equally: A Multicenter Randomized Controlled Trial of a Computer-Based Cognitive Flexibility Training. PLoS ONE 12 (3), e0172993. 10.1371/journal.pone.0172993 28257436 PMC5336244

[B191] van Heugten C. M. Ponds R. W. H. M. Kessels R. P. C. (2016). Brain Training: Hype or hope? Neuropsychological Rehabil. 26, 639–644. 10.1080/09602011.2016.1186101 27390903

[B192] van Zon L. Kirby J. R. Anderson N. (2016). The Efficacy of a Volunteer-Administered Cognitive Stimulation Program in Long-Term Care Homes. Int. Psychogeriatr. 28 (6), 995–1004. 10.1017/s1041610215002392 26804606

[B193] Vance D. E. Keltner N. L. McGuinness T. Umlauf M. G. Yuan Y. Y. (2010). The Future of Cognitive Remediation Training in Older Adults. J. Neurosci. Nurs. 42 (5), 255–256. 10.1097/jnn.0b013e3181ecb003 20968221

[B194] Vance D. E. McNees P. Meneses K. (2009). Technology, Cognitive Remediation, and Nursing: Directions for Successful Cognitive Aging. J. Gerontol. Nurs. 35 (2), 50–56. 10.3928/00989134-20090201-09 19263921

[B195] Vance D. E. (2009). The Emerging Role of Cognitive Remediation Therapy. Activities, Adaptation & Aging 33 (1), 17–30. 10.1080/01924780902718541

[B196] Vance D. Fazeli P. Shacka J. Nicholson W. McKie P. Raper J. (2017). Testing a Computerized Cognitive Training Protocol in Adults Aging with Hiv-Associated Neurocognitive Disorders: Randomized Controlled Trial Rationale and Protocol. JMIR Res. Protoc. 6 (4), e68. 10.2196/resprot.6625 28446421 PMC5422019

[B197] Vanova M. Irazoki E. García-Casal J. A. Martínez-Abad F. Botella C. Shiells K. R. (2018). The Effectiveness of ICT-Based Neurocognitive and Psychosocial Rehabilitation Programmes in People with Mild Dementia and Mild Cognitive Impairment Using GRADIOR and ehcoBUTLER: Study Protocol for a Randomised Controlled Trial. Trials 19 (100), 100–115. 10.1186/s13063-017-2371-z 29433545 PMC5810083

[B198] VanVleet T. Voss M. Dabit S. Mitko A. DeGutis J. (2018). Randomized Control Trial of Computer-Based Training Targeting Alertness in Older Adults: The ALERT Trial Protocol. BMC Psychol. 6 (22), 22–14. 10.1186/s40359-018-0233-4 29724228 PMC5934832

[B199] Vaportzis E. Martin M. Gow A. J. (2017). A Tablet for Healthy Ageing: The Effect of a Tablet Computer Training Intervention on Cognitive Abilities in Older Adults. Am. J. Geriatr. Psychiatry 25 (8), 841–851. 10.1016/j.jagp.2016.11.015 28082016 PMC5444526

[B200] Velligan D. I. Bow-Thomas C. C. Huntzinger C. Ritch J. Ledbetter N. Prihoda T. J. (2000). Randomized Controlled Trial of the Use of Compensatory Strategies to Enhance Adaptive Functioning in Outpatients with Schizophrenia. Ajp 157 (8), 1317–1328. 10.1176/appi.ajp.157.8.1317 10910797

[B201] Verghese J. Mahoney J. Ambrose A. F. Wang C. Holtzer R. (2010). Effect of Cognitive Remediation on Gait in Sedentary Seniors. Journals Gerontol. Ser. A: Biol. Sci. Med. Sci. 65A (12), 1338–1343. 10.1093/gerona/glq127 20643703

[B202] Vermeij A. Claassen J. A. Dautzenberg P. L. Kessels R. P. (2016). Transfer and Maintenance Effects of Online Working-Memory Training in normal Ageing and Mild Cognitive Impairment. Neuropsychol. Rehabil. 26 (5-6), 783–809. 10.1080/09602011.2015.1048694 26010573

[B203] Vidovich M. R. Shaw J. Flicker L. Almeida O. P. (2011). Cognitive Activity for the Treatment of Older Adults with Mild Alzheimer's Disease (AD) - PACE AD: Study Protocol for a Randomised Controlled Trial. Trials 12, 47. 10.1186/1745-6215-12-47 21329501 PMC3052177

[B204] Wang M-Y. Chang C-Y. Su S-Y. (2011). What’s Cooking? – Cognitive Training of Executive Function in the Elderly. Front. Psychol. 2 (228), 1–11. 10.3389/fpsyg.2011.00228 21954388 PMC3173828

[B205] Whitlock L. A. McLaughlin A. C. Allaire J. C. (2012). Individual Differences in Response to Cognitive Training: Using a Multi-Modal, Attentionally Demanding Game-Based Intervention for Older Adults. Comput. Hum. Behav. 28 (4), 1091–1096. 10.1016/j.chb.2012.01.012

[B206] Willis S. L. (1996). Everyday Cognitive Competence in Elderly Persons: Conceptual Issues and Empirical Findings. The Gerontologist 36 (5), 595–601. 10.1093/geront/36.5.595 8942103

[B207] Willis S. L. Tennstedt S. L. Marsiske M. Ball K. Elias J. Koepke K. M. (2006). Long-term Effects of Cognitive Training on Everyday Functional Outcomes in Older Adults. JAMA 296 (23), 2805–2814. 10.1001/jama.296.23.2805 17179457 PMC2910591

[B208] Wilson B. A. (2002). Towards a Comprehensive Model of Cognitive Rehabilitation. Neuropsychological Rehabil. 12 (2), 97–110. 10.1080/09602010244000020

[B209] Winblad B. Palmer K. Kivipelto M. Jelic V. Fratiglioni L. Wahlund L.-O. (2004). Mild Cognitive Impairment - beyond Controversies, towards a Consensus: Report of the International Working Group on Mild Cognitive Impairment. J. Intern. Med. 256 (3), 240–246. 10.1111/j.1365-2796.2004.01380.x 15324367

[B210] Withiel T. Wong D. Ponsford J. Cadilhac D. New P. Mihaljcic T. (2019). Comparing Memory Group Training and Computerized Cognitive Training for Improving Memory Function Following Stroke: A Phase II Randomized Controlled Trial. J. Rehabil. Med. 51 (5), 343–351. 10.2340/16501977-2540 30815708

[B211] Wolinsky F. D. Unverzagt F. W. Smith D. M. Jones R. Wright E. Tennstedt S. L. (2006). The Effects of the ACTIVE Cognitive Training Trial on Clinically Relevant Declines in Health-Related Quality of Life. Journals Gerontol. Ser. B: Psychol. Sci. Soc. Sci. 61 (5), S281–S287. 10.1093/geronb/61.5.s281 16960242

[B212] Wolinsky F. D. Vander Weg M. W. Martin R. Unverzagt F. W. Willis S. L. Marsiske M. (2009). Does Cognitive Training Improve Internal Locus of Control Among Older Adults? Journals Gerontol. Ser. B: Psychol. Sci. Soc. Sci. 65B (5), 591–598. 10.1093/geronb/gbp117 PMC2920943 20008028

[B213] Woods B. Aguirre E. Spector A. E. Orrell M. (2012). Cognitive Stimulation to Improve Cognitive Functioning in People with Dementia. Cochrane Database Syst. Rev. (2), CD005562. 10.1002/14651858.CD005562.pub2 22336813

[B214] Woods B. Thorgrimsen L. Spector A. Royan L. Orrell M. (2006). Improved Quality of Life and Cognitive Stimulation Therapy in Dementia. Aging Ment. Health 10 (3), 219–226. 10.1080/13607860500431652 16777649

[B215] World Health Organization (2017a). Operationalising the Concept of Intrinsic Capacity in Clinical Settings. Available from https://www.who.int/ageing/health-systems/clinical-consortium/CCHA2017-backgroundpaper-1.pdf (Retrieved October 18, 2021).

[B216] World Health Organization (2017b). 10 Facts on Ageing and Health. Available from https://www.who.int/features/factfiles/ageing/en/ (Retrieved April 19, 2019).

[B217] World Health Organization (2015). World Report on Ageing and Health. Available from https://www.who.int/ageing/publications/world-report-2015/en/ (Retrieved October 18, 2021).

[B229] Wykes T. S. van der Gaag M. (2001). Is it time to develop a new cognitive therapy for psychosis--cognitive remediation therapy (CRT)?. Clinical Psychology Review 21 (8), 1227–1256. 10.1016/S0272-7358(01)00104-0 11702514

[B218] Yam A. Marsiske M. (2013). Cognitive Longitudinal Predictors of Older Adults’ Self-Reported IADL Function. J. Aging Health 25 (8S), 163S–185S. 10.1177/0898264313495560 24385635 PMC3882335

[B219] Yamanaka K. Kawano Y. Noguchi D. Nakaaki S. Watanabe N. Amano T. (2013). Effects of Cognitive Stimulation Therapy Japanese Version (CST-J) for People with Dementia: A Single-Blind, Controlled Clinical Trial. Aging Ment. Health 17 (5), 579–586. 10.1080/13607863.2013.777395 23550665 PMC3935224

[B220] Yan L. Li L. Qing X. Zhi H. Lin L. Lei G. (2016). Cognitive Training in Older Adults with Mild Cognitive Impairment. Biomed. Environ. Sci. 29 (5), 356–364. 27353710 10.3967/bes2016.046

[B221] Yasini M. Marchand G. (2016). Adoption and Use of a mobile Health Application in Older Adults for Cognitive Stimulation. Stud. Health Technol. Inform. 221, 13–17. 10.3233/978-1-61499-633-0-13 27071867

[B222] Young D. K.-W. (2020). Multicomponent Intervention Combining a Cognitive Stimulation Group and Tai Chi to Reduce Cognitive Decline Among Community-Dwelling Older Adults with Probable Dementia: A multi-center, Randomized Controlled Trial. Dementia 19 (6), 2073–2089. 10.1177/1471301218814637 30486656

[B223] Young D. K.-w. Ng P. Y.-n. Kwok T. Ho F. Cheng D. Mak V. (2019). The Effects of an Expanded Cognitive Stimulation Therapy Model on the Improvement of Cognitive Ability of Elderly with Mild Stage Dementia Living in a Community - a Randomized Waitlist Controlled Trial. Aging Ment. Health 23 (7), 855–862. 10.1080/13607863.2018.1471586 29781725

[B224] Yuill N. Hollis V. (2011). A Systematic Review of Cognitive Stimulation Therapy for Older Adults with Mild to Moderate Dementia: An Occupational Therapy Perspective. Occup. Ther. Int. 18 (4), 163–186. 10.1002/oti.315 21425381

[B225] Zajac-Lamparska L. Trempała J. (2016). Effects of Working Memory and Attentional Control Training and Their Transfer onto Fluid Intelligence in Early and Late Adulthood. Health Psychol. Rep. 4 (1), 41–53. 10.5114/hpr.2016.56846

[B226] Zelinski E. M. (2009). Far Transfer in Cognitive Training of Older Adults. Restorative Neurol. Neurosci. 27 (5), 455–471. 10.3233/rnn-2009-0495 PMC4169295 19847070

[B227] Zelinski E. M. Spina L. M. Yaffe K. Ruff R. Kennison R. F. Mahncke H. W. (2011). Improvement in Memory with Plasticity-Based Adaptive Cognitive Training: Results of the 3-month Follow-Up. J. Am. Geriatr. Soc. 59 (2), 258–265. 10.1111/j.1532-5415.2010.03277.x 21314646

[B228] Zimmermann N. Netto T. M. Amodeo M. T. Ska B. Fonseca R. P. (2014). Working Memory Training and Poetry-Based Stimulation Programs: Are There Differences in Cognitive Outcome in Healthy Older Adults? Nre 35 (1), 159–170. 10.3233/nre-141104 24990015

