# Corrigendum: A conceptual view of cognitive intervention in older adults with and without cognitive decline—a systemic review

**DOI:** 10.3389/fragi.2024.1422949

**Published:** 2024-05-14

**Authors:** Liliana Mendes, Joana Oliveira, Fernando Barbosa, Miguel Castelo-Branco

**Affiliations:** ^1^ Coimbra Institute for Clinical and Biomedical Research, Faculty of Medicine, University of Coimbra, Coimbra, Portugal; ^2^ Faculty of Psychology and Education Science, University of Porto, Porto, Portugal

**Keywords:** brain training, cognitive stimulation, cognitive training, cognitive rehabilitation, older adults

In the published article, the Funding statement was mistakenly not included. The correct Funding statement appears below.

