# Supplementary material for: A Conceptual View of Cognitive Intervention in Older Adults With and Without Cognitive Decline—A Systemic Review
Source: Front Aging. 2022 Mar 24;3:844725. doi: 10.3389/fragi.2022.844725 (PMC9261456; doi:10.3389/fragi.2022.844725)
Supplement: Supplementary file 1 [file DataSheet1.pdf]

Appendix 1

Table: Type of cognitive intervention for Older Adults

Author(s)Year	***Cognitive intervention***	***Subjects***	***Research design***	***Duration (Month, week) and total sessions***	***Main intervention***
Velligan et al., 2000	Compensatory Cognitive training	45 individuals with psychotic disorders	Randomized controlled and follow participants out 9 months post intervention	9 months; no description of the total sessions	Apathy *vs* disinhibition, and executive functions Compensatory strategies - neuropsychological, behavioral and occupational therapy
Troyer et al., 2008	Compensatory Cognitive training	68 individuals with amnestic MCI	Randomized controlled and follow participants out 3 months post intervention	6 months; 10 total sessions	Memory External memory compensatory strategy
Twamley et al., 2012	Compensatory Cognitive training	69 individuals with psychotic disorders	Randomized controlled and follow participants out 3 months post intervention	12 weeks; 12 total sessions	Prospective memory, attention and vigilance, learning and memory, executive functioning
Greenaway et al., 2013	Compensatory Cognitive training	40 individuals with amnestic MCI	Randomized controlled and follow participants out 6 months post intervention	6 weeks; 12 total sessions	Memory and ADL External memory compensatory strategy
Schmiedek et al., 2010	Enrichment	101 adults and 103 older adults	Randomized controlled trial	6 months; 100 total sessions	Internet-based training *COGITO* (perceptual speed, episodic memory and working memory)
Linde & Alfermann, 2014	Enrichment	70 healthy older adults	Randomized controlled and follow participants out 5 months post intervention	16 weeks; physical intervention – 32 sessions, cognitive intervention – 16 sessions	Combined physical plus cognitive intervention (short-term memory, visuospatial, processing speed, concentration and logical reasoning) in healthy older adults (does not include computerized tasks)
Best et al., 2018	Enrichment	72 stroke older adults	Randomized controlled and follow participants out 6 months post intervention	26 weeks; 52 total sessions	Combined physical, social and cognitive enrichment program efficiently improve cognitive function (learning and memory, processing speed, attention, working memory, and executive functions) *Lumosity* training programme

Amnestic MCI criteria - Memory complaint; objective memory impairment for age; essentially preserved general cognitive function; largely intact functional activities; not demented (Petersen, 2004); preserved basic activities of daily living or minimal impairment in complex instrumental functions (Winblad et al., 2004)
MCI - Mild Cognitive Impairment
ADL – Activities of Daily Living

Type of cognitive intervention for Older Adults (cont.)

Author(s)Year	***Cognitive intervention***	***Subjects***	***Research design***	***Duration (Month, week) and total sessions***	***Main intervention***
Verghese et al., 2010	Cognitive Remediation	24 older adults	Randomized controlled trial	8 weeks; 72 total sessions	Processing speed, attention and executive function training Computerized cognitive remediation *Mindfit*
Mohlman et al., 2011	Cognitive Remediation	16 patients with PD	Pre and post-test design	1 month; 4 total sessions	Sustained, selective, alternating, and divided attentional abilities APT intervention (do not include computerized tasks)
Morimoto et al., 2014	Cognitive Remediation	44 older adults with major depression	Randomized controlled and follow participants out 3 months post intervention	4 weeks; 30 hours	Executive function Computer-based training
Mohlman et al., 2017	Cognitive Remediation	14 patients with PD	Pre and post-test design	10 weeks; 10 total sessions	Anxiety and executive functions Combined CBT and APT (do not include computerized tasks)
Vance et al., 2017	Cognitive Remediation	528 patients (264 with HAND and 264 without HAND)	Randomized controlled and follow participants out 1- and 2-years post intervention	10 weeks; speed of processing training groups – 10 sessions and 20 sessions; 10 sessions - contact-control condition	Processing speed Computer-based cognitive training: *ACTIVE Study - Sweep Seeker, Bird Safari, Target Tracker, Master Gardener* and *Double decision*
Choi et al., 2018	Cognitive Remediation	57 patients with schizophrenia and MCI	Randomized controlled trial	3 months, 24 total sessions	Attention, memory and executive function training *PSSCogRehab software program*

MCI - Mild Cognitive Impairment
PD – Parkinson disease
HAND - HIV-associated neurocognitive disorders
CBT - Cognitive– behavioral therapy
APT - attention process training intervention

Type of cognitive intervention for Older Adults (cont.)

Author(s)Year	***Cognitive intervention***	***Subjects***	***Research design***	***Duration (Month, week) and total sessions***	***Main intervention***
Spector et al., 2003	Cognitive Stimulation	201 older adults with dementia (115 intervention group and 86 control group)	Randomized controlled trial	7 weeks; 14 total sessions	Benefit cognition and QoL (do not include computerized tasks)
Woods et al., 2006	Cognitive Stimulation	201 older adults with dementia	Randomized controlled trial	7 weeks, 14 total sessions	QoL, cognition, dementia, mood, dependency and communication (do not include computerized tasks)
Tranter & Koutstaal, 2008	Cognitive Stimulation	44 healthy older adults (intervention and control group)	Randomized controlled trial	10-12-weeks; 15 total sessions	Fluid intelligence performance (do not include computerized tasks)
Niu et al., 2010	Cognitive Stimulation	32 older adults with AD (intervention and control group)	Randomized controlled trial	10 weeks; 20 total sessions	Executive functions and working memory (do not include computerized tasks)
Spector et al., 2010	Cognitive Stimulation	201 older adults with dementia (115 treatment and 86 control groups)	Randomized controlled trial	No description of the duration; 14 total sessions	Memory, praxis and language (do not include computerized tasks)
Vidovich et al., 2011	Cognitive Stimulation	128 patients with AD	Randomized controlled and follow participants out 6 months post intervention	12 weeks; 12 total sessions	Reducing score on the Alzheimer Disease Assessment Scale-Cognitive; QoL, mood, memory, language, executive functions, independent living abilities and psychiatric symptoms (do not include computerized tasks)
Fernández-Prado et al., 2012	Cognitive Stimulation	104 older adults (53 intervention group and 51 control group)	Randomized controlled trial	9 months; 72 total sessions	QoL and cognitive changes (do not include computerized tasks)

QoL – Quality of life
AD – Alzheimer disease

Type of cognitive intervention for Older Adults (cont.)

Author(s)Year	***Cognitive intervention***	***Subjects***	***Research design***	***Duration (Month, week) and total sessions***	***Main intervention***
Miranda-Castillo et al., 2012	Cognitive Stimulation	22 older adults with AD (intervention and control group)	Randomized controlled and follow participants out 7 weeks post intervention	7 weeks; 14 total sessions	Cognition, QoL, functional ability (do not include computerized tasks)
Moro et al., 2012	Cognitive Stimulation	30 older adults with MCI (2 groups)	Pre and post-test design	6 months; 32 total sessions	Attention, memory and executive functions (do not include computerized tasks)
Thiel et al., 2012	Cognitive Stimulation	159 older adults (114 intervention group and 45 control group)	Randomized controlled and follow participants out 6 months post intervention	2 months; 8 total sessions	Combined physical plus cognitive intervention in older adults (do not include computerized tasks)
Yamanaka et al., 2013	Cognitive Stimulation	56 older adults with dementia (26 treatment group and 30 control group)	Randomized controlled trial	7 weeks; 14 total sessions	QoL, cognition and mood (do not include computerized tasks)
Alves et al., 2014	Cognitive Stimulation	20 older adults with cognitive impairment	Randomized controlled trial	6 weeks; 17 total sessions	Changes in neuropsychological, functionality, QoL, and caregiver outcomes (do not include computerized tasks)
Dannhauser et al., 2014	Cognitive Stimulation	67 older adults with MCI	Randomized controlled trial	12 weeks; 76 total sessions	No computerized tasks - *ThinkingFit* programme (physical activity, group-based and individual cognitive stimulation - attention, processing speed, working memory, problem solving and reasoning)

AD – Alzheimer disease
QoL – Quality of life
MCI - Mild Cognitive Impairment

Type of cognitive intervention for Older Adults (cont.)

Author(s)Year	***Cognitive intervention***	***Subjects***	***Research design***	***Duration (Month, week) and total sessions***	***Main intervention***
De Oliveira et al., 2014	Cognitive Stimulation	42 healthy older adults (25 institutionalized and 17 noninstitutionalized)	Pre and post-test design	24 weeks; 48 total sessions	Memory and language (do not include computerized tasks)
Suzuki et al., 2014	Cognitive Stimulation	58 older adults (intervention and control group)	Randomized controlled trial	12 weeks; 12 total sessions	Memory, executive functions and attention (do not include computerized tasks)
Zimmermann et al, 2014	Cognitive Stimulation	14 older adults	Pre and post-test design	6 weeks; 12 total sessions	Differences between direct and indirect stimulation of WM (do not include computerized tasks)
Moro et al., 2015	Cognitive Stimulation	30 older adults with MCI (2 groups)	Pre and post-test design	6 months; 32 total sessions	General functions, executive functions, memory and language (do not include computerized tasks)
Malvy 2016	Cognitive Stimulation	11 older adults with moderate cognitive impairment	Descriptive study	12 months; 48 total sessions	Attention, memory, language, praxis, visuospatial skills and executive function training Computerized cognitive stimulation *KODRO software*
Yasini & Marchand, 2016	Cognitive Stimulation	15 older adults	Pre and post-test design	6 months; 29 minutes per day	WM, attention, concentration, visuospatial memory Tablet application *Stim'Art*
Zon et al., 2016	Cognitive Stimulation	53 older adults (37 experimental and 16 control group)	Pre and post-test design	8 weeks; 24 total sessions	Reasoning, attention and memory (do not include computerized tasks)
Castel et al., 2017	Cognitive Stimulation	176 older adults (123 intervention group and 53 control group)	Pre and post-test design	9 weeks; 18 total sessions	No computerized tasks (orientation, memory, language, gnosis, praxis, reminiscences, numerical calculation, attention, and concentration)
Capotosto et al., 2017	Cognitive Stimulation	39 older adults with mild to moderate dementia	Randomized controlled trial	7 weeks; 14 total sessions	Cognitive functioning, QoL, mood, functional activities in daily living and behavior (do not include computerized tasks)

WM – Working memory
MCI - Mild Cognitive Impairment
QoL – Quality of life

Type of cognitive intervention for Older Adults (cont.)

Author(s)Year	***Cognitive intervention***	***Subjects***	***Research design***	***Duration (Month, week) and total sessions***	***Main intervention***
Djabelkhir et al., 2017	Cognitive Stimulation	19 older adults with MCI (9 CCS and 10 CCE)	Randomized controlled trial	3 months; 12 total sessions	Computerized cognitive stimulation (*KODRO*) Global cognitive function, executive functions, working memory, episodic memory and psychosocial functioning
Dethlefs et al., 2017	Cognitive Stimulation	23 older adults (13 healthy and 10 with dementia)	Pilot nonrandomized	20 minutes, one session	Memory and communication Computer-based assistive - *Wizard-of-Oz*
Grimaud et al., 2017	Cognitive Stimulation	67 older adults	Pre and post-test design	10 weeks; 10 total sessions	Processing speed, working memory, executive functions, memory span and self-steem QoL (do not include computerized tasks)
Herrera et al., 2017	Cognitive Stimulation	37 older adults (23 experimental group and 14 control group)	Pre and post-test design	1 month; 10 total sessions	Memory, orientation, information about daily events and arithmetic (do not include computerized tasks)
Ordonez et al., 2017	Cognitive Stimulation	124 older adults (102 experimental group and 22 control group)	Pre and post-test design	No description of the duration; 12 total sessions	Global cognition, depressive and anxiety symptoms, memory complaints and learning satisfaction Electronic games equipment *Actively Station*
Piras et al., 2017	Cognitive Stimulation	35 older adults with mild-to-moderate VaD	Randomized controlled trial	7 weeks; 14 total sessions	Working memory, language, QoL, mood and behavior, everyday life functioning (do not include computerized tasks)
Stewart et al., 2017	Cognitive Stimulation	40 older adults with dementia	Pre and post-test design	7 weeks; 14 total sessions	Cognition, QoL and depression (do not include computerized tasks)

MCI - Mild Cognitive Impairment
VaD – Vascular dementia
CCS - computerized cognitive stimulation
CCE - computerized cognitive engagement
QoL – Quality of life

Type of cognitive intervention for Older Adults (cont.)

Author(s)Year	***Cognitive intervention***	***Subjects***	***Research design***	***Duration (Month, week) and total sessions***	***Main intervention***
Djabelkhir-Jemmi et al., 2018	Cognitive Stimulation	51 older adults with MCI	Pre and post-test design and follow participants out 3 months post intervention	3 months; 24 total sessions	Executive functions, attention, and processing speed Computerized cognitive stimulation *KODRO software*
Martínez-Alcalá et al., 2018	Cognitive Stimulation	22 older adults	Pilot nonrandomized	12 weeks; 24 total sessions	Attention, memory, comprehension, perception and visuospatial processes Mobile app *iBeni*
Young, 2020	Cognitive Stimulation	80 older adults (41 treatment group and 39 control group)	Randomized controlled trial	7 weeks; 14 total sessions	Cognitive ability and QoL (do not include computerized tasks)
Karssemeijer et al., 2019	Cognitive Stimulation	115 older adults with dementia	Randomized controlled and follow participants out 6 months post intervention	12 weeks; 36 total sessions	Executive function, episodic memory, working memory and psychomotor speed Interactive virtual bike tours: *Bike Labyrinth*
Valdés et al., 2019	Cognitive Stimulation	49 older adults with MCI (24 treatment group and 25 control group)	Randomized controlled trial	5 weeks; 10 total sessions	Processing speed *Road Sign Test* computer training
Young et al., 2019	Cognitive Stimulation	101 older adults with dementia (51 treatment group and 50 control group)	Randomized controlled trial	7 weeks; 14 total sessions	Cognitive functioning (do not include computerized tasks)

MCI – Mild cognitive impairment
QoL – Quality of life

Type of cognitive intervention for Older Adults (cont.)

Author(s)Year	***Cognitive intervention***	***Subjects***	***Research design***	***Duration (Month, week) and total sessions***	***Main intervention***
Power et al., 2011	Brain Training	40 older adults	Pre and post-test design	2 months; 5-10 minutes per day	Numerical ability, self-reported memory and intelligence Video game *Nintendo DS Lite Games Console* and game *Dr Kawashima´s Brain Training: How Old is Your Brain?*
McDougall & House, 2012	Brain Training	41 older adults	Pre and post-test design	6 weeks; 2–3 times per week	Perceptions of cognitive functioning and quality of life Video game *Nintendo DS Brain Training*
Nouchi et al., 2012	Brain Training	28 older adults (intervention and control group)	Randomized controlled trial	4 weeks; 20 total sessions (15 minutes)	Global cognitive status, executive functions, attention and processing speed Video game training (*Brain Age and Tetris*)
Mayas et al., 2014	Brain Training	27 older adults	Randomized controlled trial	10-12 weeks; 20 total sessions	Problem solving, mental calculation, working memory and attention Brain training application *Lumosity*
Ballesteros et al., 2015	Brain Training	40 healthy older adults	Randomized controlled and follow participants out 3 months post intervention	10-12 weeks; 20 total sessions	Attention, processing speed, visual memory and subjective wellbeing Brain training application *Lumosity*
Ballesteros et al., 2017	Brain Training	75 healthy older adults	Randomized controlled and follow participants out 6 months post intervention	10-12 weeks; 16 total sessions	Attention and spatial working memory Brain training application *Lumosity*
Van de Ven et al., 2017	Brain Training	97 older adults with cognitive impairments (3 groups)	Randomized controlled trial	12 weeks; 58 total sessions	Working memory, attention and reasoning Computerized tasks - *wwwbraingymmercom*

Type of cognitive intervention for Older Adults (cont.)

Author(s)Year	***Cognitive intervention***	***Subjects***	***Research design***	***Duration (Month, week) and total sessions***	***Main intervention***
Auffray & Juhel, 2001	Cognitive Training	82 older adults (64 experimental group and 18 control group)	Randomized controlled and follow participants out 6/9 months post intervention	No description of the duration; 6 total sessions	Attention, memory, reasoning (do not include computerized tasks)
Koltai et al., 2001	Cognitive Training	24 older adults with mild to moderate dementia (treatment and control group)	Randomized controlled and follow participants out 2-weeks post intervention	5 weeks; 6 total sessions	Mood and memory (do not include computerized tasks)
Ball et al., 2002	Cognitive Training	2832 older adults	Randomized controlled trial	5-6 weeks; 10 total sessions	Memory, reasoning (Behavioral interventions) and processing speed Computerized tasks – *ACTIVE Study*
Günther et al., 2003	Cognitive Training	19 older adults	Longitudinal design	14 weeks; 14 total sessions	Information processing speed, short-term memory, long-term memory, interference tendency, learning Computerized tasks – *Cognition I*
Belleville et al., 2006	Cognitive Training	45 older adults (28 with MCI and 17 healthy)	Pre and post-test design	8 weeks; 9 total sessions	Episodic memory and attention Computer-based training
Farina et al., 2006	Cognitive Training	32 older adults with mild to moderate dementia	Longitudinal design	6 weeks; 15 total sessions	Attention, memory, language, visuospatial abilities and categorization (do not include computerized tasks)
Margrett & Willis, 2006	Cognitive Training	98 older adults (30 in individual, 34 in collaborative training and 34 in control group)	Randomized controlled and follow participants out 6-weeks post intervention	No description of the duration; 10 total sessions	Inductive reasoning training Computerized tasks – *ACTIVE Study*
Willis et al., 2006	Cognitive Training	2832 older adults (3 treatment group and 1 control group)	Randomized controlled trial	No description of the duration; 10 total sessions	Memory, reasoning or processing speed Computerized tasks – *ACTIVE Study*

MCI – Mild Cognitive Impairment

Type of cognitive intervention for Older Adults (cont.)

Author(s)Year	***Cognitive intervention***	***Subjects***	***Research design***	***Duration (Month, week) and total sessions***	***Main intervention***
Wolinsky et al., 2006	Cognitive Training	2147 older adults	Randomized controlled and follow participants out 24 months post intervention	No description of the duration; 10 total session	Memory, inductive reasoning and processing speed Computerized tasks – *ACTIVE Study*
Basak et al., 2008	Cognitive Training	40 older adults (experimental and control group)	Randomized controlled and follow participants out 7/8-week post intervention	4-5 weeks; 15 total sessions	Executive functions (working memory, short-term memory, switching, inhibition and reasoning) Video game-based – *Rise of Nations*
Barnes et al., 2009	Cognitive Training	47 older adults with MCI (intervention and control group)	Randomized controlled trial	6 weeks; 30 total sessions	Processing speed and working memory Computer-based cognitive training
Navarro et al., 2009	Cognitive Training	36 older adults (software group; paper and pencil group; and mixed group)	Randomized controlled trial	No description of the duration; 3 groups received >38 total sessions; and 3 Groups received <38 total sessions	Cognitive and memory function Computer-based program -*How to Improve Your Mental Skills* and paper and pencil program
Park et al., 2009	Cognitive Training	129 older adults	Randomized controlled trial	12 weeks; 12 total sessions	Cognitive function and depressive symptoms (do not include computerized tasks)
Wolinsky et al., 2009	Cognitive Training	1534 older adults (4 groups)	Randomized controlled and follow participants out 5-years post intervention	6 weeks; 10 total sessions	Memory, reasoning and processing speed Computerized tasks – *ACTIVE Study*
Ball et al., 2010	Cognitive Training	908 older adults	Randomized controlled and follow participants out 6-years post intervention	5-6 weeks; 10 total sessions	Memory, reasoning and processing speed Computerized tasks – *ACTIVE Study*

MCI – Mild Cognitive Impairment

Type of cognitive intervention for Older Adults (cont.)

Author(s)Year	***Cognitive intervention***	***Subjects***	***Research design***	***Duration (Month, week) and total sessions***	***Main intervention***
Borella et al., 2010	Cognitive Training	40 older adults (intervention and control group)	Randomized controlled and follow participants out 8 months post intervention	2 weeks; 3 total sessions	Verbal and visuospatial working memory, short-term memory, inhibition, processing speed, and fluid intelligence (include computerized and paper and pencil tasks)
Finn & McDonald, 2011	Cognitive Training	25 older adults with MCI	Randomized controlled trial	11 weeks; 30 total sessions	Attention, processing speed, visual memory and executive functions Computerized cognitive training, *Lumosity*
Gross et al., 2011	Cognitive Training	2802 older adults (4 groups)	Randomized controlled trial	6 weeks; no description of the total sessions	Memory, reasoning and global mental status - *ACTIVE Study* (include computerized tasks – processing speed)
Irigaray et al., 2011	Cognitive Training	76 older adults (experimental and control group)	Randomized controlled trial	12 weeks; 12 total sessions	Attention, memory, executive function, QoL and psychological well-being - paper and pencil tasks
Paris et al., 2011	Cognitive Training	46 older adults with PD (16 experimental and 12 control group)	Randomized controlled trial	4 weeks; 12 total sessions	Attention, working memory, memory, psychomotor speed, executive functions, and visuospatial abilities, language, calculations skills, and culture Computer-based program (*SmartBrain*) and paper and pencil tasks
Wang et al., 2011	Cognitive Training	57 older adults (31 experimental group and 26 control group)	Randomized controlled and follow participants out 6-weeks post intervention	5 weeks; 5 total sessions	Executive function, prospective memory, planning and working memory (include computerized tasks)
Zelinski et al., 2011	Cognitive Training	487 older adults (242 experimental group and 245 control group)	Randomized controlled and follow participants out 3 months post intervention	8-10 weeks; 40 total sessions	Processing speed and auditory processing (include computerized tasks)

MCI – Mild Cognitive Impairment
QoL – Quality of life
PD – Parkinson disease

Type of cognitive intervention for Older Adults (cont.)

Author(s)Year	***Cognitive intervention***	***Subjects***	***Research design***	***Duration (Month, week) and total sessions***	***Main intervention***
Cheng et al., 2012	Cognitive Training	270 healthy older adults (3 groups)	Randomized controlled and follow participants out 6/12-months post intervention	12 weeks; 24 total sessions	Memory, reasoning, problem solving strategies, visuospatial construction No computerized tasks
Herrera et al., 2012	Cognitive Training	22 adults with A-MCI (experimental and control group)	Randomized controlled and follow participants out 6 months post intervention	12 weeks; 24 total sessions	Memory and attention (include computerized tasks)
Jackson et al., 2012	Cognitive Training	183 older adults (intervention and control group)	Randomized controlled trial	16 weeks; 160 total hours	Inductive reasoning No computerized tasks
Irigaray et al., 2012	Cognitive Training	76 older adults (experimental and control group)	Randomized controlled trial	12 weeks 12 total sessions	Attention, memory and executive functions training No computerized tasks
Simpson et al., 2012	Cognitive Training	34 older adults (intervention and control group)	Randomized controlled and follow participants out three-weeks post intervention	4 weeks; 21 total sessions	Reaction time, inspection time, short-term memory, executive function, visuospatial acuity, arithmetic, visuospatial memory, visual scanning and working memory Computerized training: *mybraintrainercom*
Whitlock et al., 2012	Cognitive Training	39 older adults (intervention and control group)	Pre and post-test design	2 weeks; 14 total sessions	Spatial ability, executive function and memory *World of Warcraft game*
Naismith et al., 2013	Cognitive Training	50 older adults with PD (35 intervention group, 15 control group)	Single-blinded waitlist control design	7 weeks; 14 total sessions	Memory and psychoeducation Computer-based training program *(Neuropsychological Educational Approach to Remediation-NEAR)*

A-MCI – Amnestic Mild Cognitive Impairment
PD - Parkinson disease

Type of cognitive intervention for Older Adults (cont.)

Author(s)Year	***Cognitive intervention***	***Subjects***	***Research design***	***Duration (Month, week) and total sessions***	***Main intervention***
Ball et al., 2013	Cognitive Training	2802 older adults (4 groups)	Randomized controlled trial	No description of the duration; 10 total sessions	Processing speed *ACTIVE Study* (include computerized tasks – SOPT speed of processing)
Carretti et al., 2013	Cognitive Training	40 older adults (experimental and control group)	Randomized controlled and follow participants out 6 months post intervention	2 weeks; 3 total sessions	Verbal working memory, language comprehension and reasoning (include paper and pencil tasks)
Garcia-Campuzano et al., 2013	Cognitive Training	24 older adults (13 intervention group and 11 control group)	Randomized controlled trial	8 weeks; 24 total sessions	Memory (include computerized tasks)
Gigler et al., 2013	Cognitive Training	18 older adults (11 cognitively healthy and 7 MCI)	Pre and post-test design	10 weeks; 17 total sessions	General cognitive function Computerized tasks – *CogniFit*
González-Palau et al., 2013	Cognitive Training	33 with dementia; 52 with MCI; 95 healthy older adults	Exploratory research	12 weeks; 36 total sessions	Perception, attention, episodic memory, and working memory *Gradior software*
Jones et al., 2013	Cognitive Training	1659 older adults	Randomized controlled and follow participants out 5-years post intervention	5-6 weeks; 10 total sessions	Memory, reasoning and processing speed Computerized tasks – *ACTIVE Study*
Kwok et al., 2013	Cognitive Training	176 older adults (intervention and control group)	Randomized controlled trial	8 weeks; 8 total sessions	Cognitive function and QoL (do not include computerized tasks – *Active Mind*)
Maseda et al., 2013	Cognitive Training	101 older adults with and without memory impairments (3 groups)	Pre and post-test design	24 weeks; 72 total sessions	Memory attention, language, calculation, abstract reasoning, perception, orientation and praxis Computerized cognitive training – *Telecognitio*

MCI - Mild Cognitive Impairment
QoL – Quality of life

Type of cognitive intervention for Older Adults (cont.)

Author(s)Year	***Cognitive intervention***	***Subjects***	***Research design***	***Duration (Month, week) and total sessions***	***Main intervention***
Netto et al., 2013	Cognitive Training	20 older adults (11 experimental group and 9 control group)	Randomized controlled trial	3 months; 144 total sessions	Working memory Paper and pencil tasks
Rebok et al., 2013	Cognitive Training	2802 older adults (4 groups)	Randomized controlled and follow participants out 5-years post intervention	No description of the duration; 10 total sessions	Memory, inductive reasoning and processing speed Computerized tasks – *ACTIVE Study*
Rojas et al., 2013	Cognitive Training	46 older adults with MCI (24 training group and 22 control group)	Randomized controlled and follow participants out 1-year post intervention	6 months; 48 total sessions	Cognitive function (do not include computerized tasks)
Shatil, 2013	Cognitive Training	122 older adults (4 groups)	Randomized controlled trial	16 weeks; 48 total sessions	General cognitive function Computerized tasks – *CogniFit*
Sisco et al., 2013	Cognitive Training	1912 older adults	Randomized controlled trial	5-6 weeks; 10 total sessions	Memory, reasoning and processing speed Computerized tasks – *ACTIVE Study*
Feng et al., 2014	Cognitive Training	151 healthy older adults (90 intervention group and 61 control group)	Randomized controlled and follow participants out 5-years post intervention	12 weeks; 24 total sessions	Reasoning, memory and problem solving and behavioral exercises (do not include computerized tasks)
González-Palau et al., 2014	Cognitive Training	50 older adults (11 with MCI and 39 healthy)	Pre and post-test design	12 weeks; 36 total sessions	Perception, attention, episodic memory, and working memory *Gradior software*
Kanaan et al., 2014	Cognitive Training	21 older adults with AD	Randomized controlled and follow participants out 2/4 months post intervention	2 weeks; 40-50 total hours	Working memory, sustained, divided and switching attention Computerized and paper and pencil training tasks

MCI – Mild Cognitive Impairment
AD - Alzheimer´s disease

Type of cognitive intervention for Older Adults (cont.)

Author(s)Year	***Cognitive intervention***	***Subjects***	***Research design***	***Duration (Month, week) and total sessions***	***Main intervention***
Petrelli et al., 2014	Cognitive Training	65 older adults with PD (3 groups)	Randomized controlled trial	6 weeks; 12 total sessions	Depression, QoL Attention, memory and executive functions – *NEUROvitalis* Attention, memory, language and creative thinking -*Mentally Fit* Do not include computerized tasks
Rebok et al., 2014	Cognitive Training	2832 adults (4 groups)	Randomized controlled and follow participants out 10 years post intervention	5-6 weeks; 10 total sessions	Memory, reasoning and processing speed Computerized tasks – *ACTIVE Study*
Peña et al., 2014	Cognitive Training	44 older adults with PD (experimental and control group)	Randomized controlled trial	3 months; 36 total sessions	Attention, memory, processing speed, language, executive functioning, social cognition and ADL *REHACOP* - paper and pencil training tasks
Eggenberger et al., 2015	Cognitive Training	71 older adults (3 groups)	Randomized controlled and follow participants out 3 and 6 months post intervention	6 months; 48 total sessions	Physical training, memory and simultaneous cognitive–physical training (include video game dancing and computerized tasks)
Candela et al., 2015	Cognitive Training	72 older adults (3 groups)	Randomized controlled trial	4 months; 16 total sessions	Long-term memory and selective attention Not computerized tasks
Cohen-Mansfield et al., 2015	Cognitive Training	44 older adults with memory complaints (3 groups)	Randomized controlled trial	10 weeks; no description of the total sessions	Cognitive function and memory *ACTIVE Study* (do not include computerized tasks)
Gooding et al., 2015	Cognitive Training	74 older adults with cognitive decline (3 groups)	Randomized controlled trial	16 weeks; 32 total sessions	Memory, attention and executive function Computerized cognitive training: *BrainFitness*
Leung et al., 2015	Cognitive Training	209 older adults	Randomized controlled trial	13 weeks; 39 total sessions	Attention and working memory Computerized cognitive training: *BrainFitness*
Millán-Calenti et al., 2015	Cognitive Training	160 older adults (experimental and control group)	Randomized controlled trial		Cognitive function and depressive symptomatology Computerized cognitive training – *Telecognitio*

PD - Parkinson disease
QoL – Quality of life
ADL – Activities of Daily Living

Type of cognitive intervention for Older Adults (cont.)

Author(s)Year	***Cognitive intervention***	***Subjects***	***Research design***	***Duration (Month, week) and total sessions***	***Main intervention***
Petrelli et al., 2015	Cognitive Training	47 older adults with PD (3 groups)	Randomized controlled and follow participants out 1-year post intervention	6 weeks; 12 total sessions	Attention, memory, and executive functions – *NEUROvitalis*; Attention, memory, executive functions and language - *Mentally fit* Do not include computerized tasks
Amieva et al., 2016	Cognitive Training	653 older adults with AD (4 groups)	Randomized controlled trial	24 months; 18 total sessions	Memory, attention, language, executive function Do not include computerized tasks
Chan et al., 2016	Cognitive Training	54 older adults (3 groups)	Randomized controlled trial	3 months; 180 total hours	Processing speed, mental control, episodic memory, visuospatial processing Include paper and pencil and computerized tasks – *iPad*
Binder et al., 2016	Cognitive Training	84 older adults (4 groups)	Randomized controlled and follow participants out 6 months post intervention	10 weeks; 50 total sessions	Executive functions, attentional control, working memory and speed Computerized tasks – *iPad*
De Luca et al., 2016	Cognitive Training	20 older adults with dementia (experimental and control group)	Randomized controlled trial	8 weeks; 24 total sessions	Attention process, visuospatial memory, verbal fluencies and praxis abilities Computerized and paper and pencil tasks
Giuli et al., 2016	Cognitive Training	321 older adults with mild-moderate AD , MCI and healthy (experimental and control group)	Randomized controlled trial	10 weeks; 10 total sessions	MCI and AD subjects: attention and visuospatial processes<br>Healthy: working memory and Learning processes Not computerized tasks
Küster et al., 2016	Cognitive Training	54 older adults at risk of dementia (3 groups)	Randomized controlled and follow participants out 3 months post intervention	10 weeks; 50 total sessions	Memory, attention and executive functions Computerized cognitive training
Li et al., 2016	Cognitive Training	40 older adults	Randomized controlled trial	6 weeks; 16 total sessions	Executive function and memory Presented in paper and pencil, and computerized form

PD - Parkinson disease
AD - Alzheimer´s disease
MCI – Mild Cognitive Impairment

Type of cognitive intervention for Older Adults (cont.)

Author(s)Year	***Cognitive intervention***	***Subjects***	***Research design***	***Duration (Month, week) and total sessions***	***Main intervention***
Lopes & Argimon, 2016	Cognitive Training	83 older adults (45 experimental group and 38 control group)	Pre and post-test design	8 weeks; 8 total sessions	Executive functions and memory Not computerized tasks
Marusic et al., 2018	Cognitive Training	16 older adults (intervention and control group)	Randomized controlled trial	2 weeks; 12 total sessions	Executive functions, auditory and visual attention, memory, spatial skills (include computerized tasks)
Nouchi et al., 2016	Cognitive Training	64 healthy older adults (intervention and control group)	Randomized controlled trial	6 months; 115 total sessions	Executive functions, attention, episodic memory, short-term and working memory, verbal ability, processing speed Paper and pencil tasks
Ross et al., 2016	Cognitive Training	1806 older adults (3 groups)	Randomized controlled trial after 5 years post intervention	6 weeks; 10 total sessions	Processing speed and memory *ACTIVE Study* (include computerized and paper and pencil tasks)
Vermeij et al., 2016	Cognitive Training	41 older adults (18 with MCI and 23 healthy)	Randomized controlled and follow participants out 3 months post intervention	5 weeks; 25 total sessions	Working memory<br>Computer-based training: *Cogmed*
Yan et al., 2016	Cognitive Training	45 older adults with MCI (intervention group and control group)	Randomized controlled trial	2 months; no description of the total sessions	Attention, memory, cognitive, language and task performance training Do not include computerized tasks
Zajac-Lamparska & Trempała, 2016	Cognitive Training	80 healthy older adults	Randomized controlled trial	4 weeks; 7 total sessions	Working memory and attentional control (include computerized tasks)
Bellander et al., 2017	Cognitive Training	39 older adults (19 experimental group and 20 control group)	Randomized controlled trial	6 weeks; 24 total sessions	Memory (include computerized tasks)
Buitenweg et al., 2017	Cognitive Training	158 healthy older adults (3 groups)	Randomized controlled and follow participants out 4-weeks post intervention	12 weeks; 57 total sessions	Working memory, attention and reasoning and mock training Computerized tasks - *wwwbraingymmercom*
Cantarella et al., 2017	Cognitive Training	36 older adults (intervention and control group)	Randomized controlled trial	2 weeks; 3 total sessions	Working memory, reasoning (include paper and pencil tasks)

MCI – Mild Cognitive Impairment

Type of cognitive intervention for Older Adults (cont.)

Author(s)Year	***Cognitive intervention***	***Subjects***	***Research design***	***Duration (Month, week) and total sessions***	***Main intervention***
Golino et al., 2017	Cognitive Training	80 older adults (47 intervention group and 33 control group)	Randomized controlled trial	12 weeks; 12 total sessions	Episodic memory, attention, processing speed, and working memory Include computerized and paper and pencil tasks
Grönholm-Nyman et al., 2017	Cognitive Training	33 older adults (17 training group and 16 control group)	Randomized controlled and follow participants out 1-year post intervention	5 weeks; 15 total sessions	Inhibition, working memory, verbal fluency, fluid intelligence, episodic memory and visuomotor speed Include computerized tasks
Reijnders et al., 2017	Cognitive Training	376 healthy older adults (228 experimental group and 148 control group)	Randomized controlled and follow participants out 4-weeks post intervention	4 weeks; no description of the total sessions	Cognitive functioning and psychological well-being Include computerized tasks: "*Keep your brain fit!*"
Ross et al., 2017	Cognitive Training	2402 older adults (4 groups)	Randomized controlled trial after 10 years post intervention	No description of the duration; 10 total sessions	Processing speed, reasoning and memory *ACTIVE Study* (include computerized and paper and pencil tasks)
Savulich et al., 2017	Cognitive Training	42 older adults with A-MCI (training and control group)	Randomized controlled trial	4 weeks; 8 total sessions	Episodic memory and visuospatial Computerized tasks – *Game Show* on *iPad*
Souders et al., 2017	Cognitive Training	60 older adults	Randomized controlled trial	4 weeks; 23 total sessions	Reasoning, planning, spatial reasoning, processing speed, task switching, and working memory Include computerized tasks: *Mind Frontiers Game*
Vaportzis et al., 2017	Cognitive Training	43 older adults (22 intervention group and 21 control group)	Randomized controlled trial	10 weeks; 10 total sessions	Verbal comprehension, perceptual reasoning, working memory, processing speed Tablet computer tasks
Brinke et al., 2018	Cognitive Training	123 older adults (3 groups)	Randomized controlled and follow participants out 1-year post intervention	8 weeks; 24 total sessions	Focus, speed, memory, visual, problem solving and language Computerized tasks – *Fit Brains* Include physical training

A-MCI – Amnestic Mild Cognitive Impairment

Type of cognitive intervention for Older Adults (cont.)

Author(s)Year	***Cognitive intervention***	***Subjects***	***Research design***	***Duration (Month, week) and total sessions***	***Main intervention***
Brum et al., 2018	Cognitive Training	96 healthy older adults (Study 1 – 18 trained group, 28 control group and Study 2 – 23 trained group, 27 control group)	Randomized controlled and follow participants out 6 months post intervention	2 weeks; 6 total sessions	Verbal and visuospatial working memory, short-term memory, inhibition, processing speed, and fluid intelligence (include computerized and paper and pencil tasks)
Chen et al., 2018	Cognitive Training	86 healthy older adults (1 control group and 4 experimental group)	Randomized controlled trial	10 weeks; 10 total sessions	Reasoning, spatial working memory and numerical working memory Do not include computerized tasks
Fellman et al., 2018	Cognitive Training	52 older adults with PD (experimental and control groups)	Randomized controlled trial	5 weeks; 15 total sessions	Working memory Include computerized tasks
Frankenmolen et al., 2018	Cognitive Training	60 older adults with subjective memory complaints (experimental and control group)	Randomized controlled and follow participants out 6 months post intervention	7 weeks; 7 total sessions	Memory and attention (include computerized tasks - *COGPAK* and paper and pencil tasks)
Goghari & Lawlor-Savage, 2018	Cognitive Training	97 older adults (3 groups)	Randomized controlled trial	8 weeks; 40 total sessions	Working memory, logic and planning Computerized tasks -*wwwbraingymmercom*
Kalbe et al., 2018	Cognitive Training	81 healthy older adults (3 groups)	Randomized controlled and follow participants out 1-year post intervention	7 weeks; 14 total sessions	Attention, memory, and executive functions – *NEUROvitalis,* and physical training Do not include computerized tasks
Kuo et al., 2018	Cognitive Training	46 healthy older adults (3 groups)	Randomized controlled trial	8 weeks; 16 total sessions	Attentional focusing, executive functions, short-term memory and memory Do not include computerized tasks - *Card Games*
Lee et al., 2018	Cognitive Training	20 older adults with MCI and mild dementia (2 groups)	Randomized controlled trial	3 weeks; 12 total sessions	Computerized tasks - *COMCOG* and *Bettercog* (memory, attention, orientation, calculation, executive function, language, comprehension and spatiotemporal abilities)

PD - Parkinson disease
MCI – Mild Cognitive Impairment

Type of cognitive intervention for Older Adults (cont.)

Author(s)Year	***Cognitive intervention***	***Subjects***	***Research design***	***Duration (Month, week) and total sessions***	***Main intervention***
López-Higes et al., 2018	Cognitive Training	81 older adults with and without subjective cognitive decline (2 groups)	Randomized controlled and follow participants out 6 months post intervention	3 months; 30 total sessions	Cognitive status, executive functions, working memory and depressive symptomatology Do not include computerized tasks
López-Higes et al., 2018	Cognitive Training	66 older adults with and without subjective cognitive decline (2 groups)	Randomized controlled and follow participants out 6 months post intervention	10 weeks; 30 total sessions	Cognitive status, executive functions, working memory and language Do not include computerized tasks
McEwen et al., 2018	Cognitive Training	55 older adults with subjective memory impairments (2 groups)	Randomized controlled trial	4 weeks; 8 total sessions	Memory, executive functions, processing speed, attention, cognitive flexibility and physical training Include computerized tasks and stationary cycling
Nousia et al., 2018	Cognitive Training	50 older adults with early-stage AD (training and control group)	Randomized controlled trial	15 weeks; 30 total sessions	Episodic and delayed memory, attention, executive function, language and processing speed (include computerized *Rehacom software* and paper and pencil tasks)
Park & Park, 2018	Cognitive Training	78 older adults with MCI (experimental and control group)	Randomized controlled trial	10 weeks; 30 total sessions	Attention, memory, visuospatial abilities and physical training Include computerized tasks - *Nintendo Wii* and *CoTras program*
Rizkalla, 2018	Cognitive Training	56 healthy older adults (experimental and control group)	Randomized controlled trial	4 weeks; 20 total sessions	Executive functioning, memory and emotion training Paper and pencil tasks
Ross et al., 2018	Cognitive Training	1806 older adults (4 groups)	Randomized controlled trial after 5 years post intervention	6 weeks; 10 total sessions	Processing speed, reasoning and memory *ACTIVE Study* (include computerized and paper and pencil tasks)
Smith et al., 2018	Cognitive Training	351 older adults (intervention and control group)	Randomized controlled and follow participants out 6/12 months post intervention	6 weeks: 10 total sessions	Processing speed Computer-based cognitive training: *Road Tour*

AD – Alzheimer disease
MCI – Mild Cognitive Impairment

Type of cognitive intervention for Older Adults (cont.)

Author(s)Year	***Cognitive intervention***	***Subjects***	***Research design***	***Duration (Month, week) and total sessions***	***Main intervention***
Sukontapol et al., 2018	Cognitive Training	60 older adults with MCI (intervention and control group)	Pre and post-test design and follow participants out 6/12 months post intervention	3 months; 6 total sessions	Executive function, attention, memory and visuospatial perception (*TEAM-V*) Do not include computerized tasks
Tagliabue et al., 2018	Cognitive Training	83 healthy older adults (30 experimental group and 53 control group)	Pre and post-test design	3 months; 13 total sessions	Multi-domain cognitive training Do not include computerized tasks
VanVleet et al., 2018	Cognitive Training	120 older adults with age-related cognitive decline (experimental and control group)	Randomized controlled trial and follow participants out 3 months post intervention	3 months; 36 total sessions	Attention and executive functions Include computerized tasks: *TAPAT*
Nouchi et al., 2019	Cognitive Training	60 healthy older adults (intervention and control group)	Randomized controlled trial	6 weeks; 30 total sessions	Processing speed, attention, inhibition, memory Include TV-based cognitive training: *Car Driving*
Matysiak et al., 2019	Cognitive Training	85 healthy older adults (experimental and control group)	Pre and post-test design and follow participants out 3 months post intervention	5 weeks; 25 total sessions	Working memory, memory updating, inhibition, attention shifting, short-term memory and reasoning Include computerized tasks
Withiel et al., 2019	Cognitive Training	65 older adults (3 groups)	Randomized controlled trial and follow participants out 6 weeks post intervention	6 weeks; 30 total sessions	Memory Computerized cognitive training: *Lumosity vs* paper and pencil program - *Making the Most of your Memory: An Everyday Memory Skills Program*

MCI – Mild Cognitive Impairment
TAPAT – Tonic and Phasic Alertness Training

Type of cognitive intervention for Older Adults (cont.)

Author(s)Year	***Cognitive intervention***	***Subjects***	***Research design***	***Duration (Month, week) and total sessions***	***Main intervention***
Loewenstein et al., 2004	Cognitive Rehabilitation	44 older adults with AD	Randomized controlled and follow participants out 3 months post intervention	12-16 weeks; 24 total sessions	Memory and functional performance Include computer games training (memory, concentration, and problem-solving skills)
Craik et al., 2007	Cognitive Rehabilitation	49 healthy older adults	Randomized controlled trial	12 weeks; 14 total sessions	Memory (do not include computerized tasks)
Levine et al., 2007	Cognitive Rehabilitation	46 healthy older adults (2 groups)	Randomized controlled and follow participants out 6 months post intervention	4 weeks; 4 total sessions	Memory and psychosocial training Include computerized software and paper and pencil tasks
Stuss et al., 2007	Cognitive Rehabilitation	49 older adults (2 groups)	Randomized controlled and follow participants out 6 months post intervention	12 weeks; 14 total sessions	Memory, goal management and psychosocial status Paper and pencil tasks
Kurz et al., 2009	Cognitive Rehabilitation	28 older adults (18 with MCI and 10 AD)	Pre and post-test design	4 weeks; 12 total sessions	Relaxation techniques, activity planning, self-assertiveness training, stress management, external memory aids, memory training, and motor exercise (does not include computerized tasks)
Jelcic et al., 2014	Cognitive Rehabilitation	27 older adults with AD (3 groups)	Randomized controlled trial	3 months; 24 total sessions	Language, memory, attention and visuospatial abilities (include computerized and paper and pencil tasks)
Mansbach et al., 2017	Cognitive Rehabilitation	43 older adults with mild cognitive deficits (20 treatment group and 23 control group)	Pre and post-test design	3 weeks; 9 sessions	Global cognitive functioning, attention and memory Include computerized tasks: *Mansbach Health Tools – Memory Match*

AD – Alzheimer disease
MCI – Mild Cognitive Impairment

Type of cognitive intervention for Older Adults (cont.)

Author(s)Year	***Cognitive intervention***	***Subjects***	***Research design***	***Duration (Month, week) and total sessions***	***Main intervention***
Clare et al., 2018	Cognitive Rehabilitation	475 older adults with dementia (intervention and control groups)	Randomized controlled and follow participants out 3/9 months post intervention	3 months: 120 total sessions plus 4 total sessions (6 months)	Problem-solving, attention and concentration Do not include computerized tasks
Fasilis et al., 2018	Cognitive Rehabilitation	10 older adults with mild dementia	Pre and post-test design	4-5 weeks; no description of the total sessions	Working memory, attention, problem solving, motivation, organization and impulsivity<br>Computerized tasks – *Main Tasks*
Maggio et al., 2018	Cognitive Rehabilitation	20 older adults with PD (experimental group and control group)	Randomized controlled trial	8 weeks; 24 total sessions	Executive and visuospatial, memory, attention, and speech abilities Include paper and pencil and computerized tasks – *BTS-Nirvana*
Vanova et al., 2018	Cognitive Rehabilitation	400 adults with MCI and mild dementia (4 groups)	Randomized controlled and follow participants out 4 months/1-year post intervention	12 months; no description of the total sessions	Attention, language, executive functions, perception, reasoning, calculus and memory<br>Computerized tasks: *GRADIOR, ehcoBUTLER*

PD - Parkinson disease
MCI – Mild Cognitive Impairment

Methods:

This literature review was planned and critically evaluated using the databases for articles on cognitive intervention studies published between 2000 and 2019: Fonte Acadêmica, PsycArticles, PsyBooks, Psychology and Behavioral Sciences Collection, PsycINFO, and PubMed, via EBSCO. Literature searched was completed using the following terms: "cognitive rehabilitation" OR "cognitive training" OR "cognitive remediation" OR "cognitive stimulation" OR "compensatory training" OR "computerized cognitive training" OR "brain training") and ("older adults" OR “elderly”). The search was very limited to Humans and publications in English, Portuguese, Spanish, and French journals only. We also conducted a recursive search for potential studies by screening the bibliographies of all relevant studies identified. Reference list of all citations to the ultimately included articles were also searched for articles that meets inclusion criteria.

The critically evaluate the studies used we used strict inclusion criteria on the following bases: (1) Peer-reviewed publication; (2) Empirical studies involving older adults; (3) Healthy older adults and older adults with several types of dementia, with a sample size of at least ten; (4) Reported use of a neurocognitive assessment to screen for impairment; (5) Randomized or non-randomized controlled trials, with or without follow-up; (6) Use of a cognitive intervention program; (7) Pre and post-test design; (8) Exploratory and longitudinal research (Figure 1).

On the other hand, the exclusion criteria were: (1) Sample of younger adults; (2) Studies limited to the application of functional magnetic resonance imaging (fMRI) methods/techniques; (3) Studies in the field of medicine and physical rehabilitation, or where rehabilitation is limited to the pharmacological treatment or repetitive transcranial magnetic stimulation; (4) Thesis or dissertations; (5) Studies with children; (6) Case studies; (7) Systematics reviews; (8) Articles not available, even after being requested to the authors (Figure 1).

References:

Koltai, D.,Welsh-Bohmer, K., and Schmechel, D. (2001). Influence of Anosognosia on Treatment Outcome Among Dementia Patients. *Neuropsychological Rehabil. 11* (3-4), 455–475. doi:10.1080/09602010042000097

Petersen, R. C. (2004). Mild Cognitive Impairment as a Diagnostic Entity. *J. Intern. Med. 256* (3), 183–194. doi:10.1111/j.1365-2796.2004.01388.x

Winblad, B., Palmer, K., Kivipelto, M., Jelic, V., Fratiglioni, L., Wahlund, L.-O., et al. (2004). Mild Cognitive Impairment - beyond Controversies, towards a Consensus: Report of the International Working Group on Mild Cognitive Impairment. J*. Intern. Med. 256* (3), 240–246. doi:10.1111/j.1365-2796.2004.01380.x
